# A comprehensive review of titanium dioxide nanoparticles in cementitious composites

**DOI:** 10.1016/j.heliyon.2024.e39238

**Published:** 2024-10-11

**Authors:** J. Jenima, M. Priya Dharshini, M.L. Ajin, J. Jebeen Moses, Krishna Priya Retnam, Krishna Prakash Arunachalam, Siva Avudaiappan, Ramon Francisco Arrue Munoz

**Affiliations:** a PG & Research Department of Physics, Holy Cross College (Autonomous), Nagercoil, Affiliated to Manonmaniam Sundaranar University, Vishakapatnam, Tirunelveli, Tamilnadu, India; b Department of Mechanical Engineering, St. Xavier's Catholic College of Engineering (Autonomous), Chunkankadai, Nagercoil 629003, India; c Departamento de Ciencias de la Construcción, Facultad de Ciencias de la Construcción Ordenamiento Territorial, Universidad Tecnológica Metropolitana, Santiago, Chile; d Facultad de Medicina y Ciencia, Departamento de CienciasBiológicas y Químicas, Universidad San Sebastián, Lientur 1457, Concepción, Chile; e Department of Physiology, Saveetha Dental College and Hospitals, SIMATS, Chennai, 600077, India

**Keywords:** Photocatalysis, Titanium dioxide, Nanotechnology, Cementitious materials, Durability, Concrete, Construction sustainability

## Abstract

Nanomaterials are incorporated to improve the characteristics of conventional concrete materials. Among them, Titanium dioxide (TiO_2_) nanoparticles exhibit sustainable concrete features such as optimization of materials, improvement of structures, recycling, and innovative waste management in materials and processes. This paper thoroughly elaborates on the effect of TiO_2_ nanoparticles on the composites of cement which in turn modifies the physical, mechanical, and ability to resist any chemical action, climate change, and abrasion; Furthermore, this study emphasizes the small fragment size, increased surface area and photocatalytic properties of cementitious composites. The key parameters for choosing TiO_2_ nanoparticles encompass their strength, antimicrobial characteristics, and stability against external environmental factors, covering a wide range of compatibility issues and exploring the fundamental characteristics of the material imbibing in cement. The present review includes the basic features of TiO_2_ nanoparticles, their utilization in cement, the fabrication processes, and influential factors on the workability of concrete. The review also comprises the workability of fresh concrete, mechanical qualities related to compressive, flexural, and tensile strength, and durability variables such as electrical resistivity, permeability, carbonation resistance, freeze-thaw cycles, and sulfate attack resistance. The report further investigates the ideal dosage of TiO_2_ nanoparticles and compares it to other nanomaterials utilized in cementitious compositions. More importantly, it emphasizes nano-titanium dioxide's ability to deal with pollutants in urban areas, notably nitrogen oxides (NOx), via its photocatalytic characteristics. The study explains how nano-TiO_2_ affects the microstructure of cement-based materials, resulting in improved durability, performance, and mechanical characteristics. Finally, this study outlines present problems and recommends future research using nano-TiO_2_ in cement-based materials.

## 1 Introduction

Nanomaterial-based cement composites are well-known for their strong mechanical attributes and longevity [1]. Recently, there has been a search for integrating nanoparticles in conventional cementitious materials [[2], [3], [4], [5], [6]]. Inclusion of unique elements, maintaining environment-sustainable features, and augmenting intrinsic traits are to be taken into account [[7], [8], [9], [10], [11], [12]]. Cement mortar is a composite material used in the construction industry, undergoes synchronous adjustments, and provides a nanostructured composition [13]. Many challenges set limitations in understanding their functionality as structural materials. Inherent functional properties are less; there are restrictions in chemical resistance, tensile strength, cracking, and brittleness [[14], [15], [16]]. Concerning the industrial emission of CO_2_, major environmental issues in the construction sector are rising and must be tackled promptly. Approximately 5–8% of the world's anthropogenic CO_2_ emissions can be traced back to cement manufacturing [[17], [18], [19], [20], [21], [22], [23], [24]]. Innovative methodologies have been explored involving the incorporation of nanomaterials into cement-based materials. Due to its adaptable nature, mechanical properties, and widespread availability, concrete is extensively used in the infrastructure sector, resulting in a global per capita consumption of approximately 20 billion metric tons [25]. Cement is the main material in the construction industry due to its enormous usage. It is reported that producing a particular standard ton of conventional Ordinary Portland Cement (OPC) leads to significant CO_2_ emissions, contributing to an increased carbon footprint [26]. The construction industry is one of the sectors most associated with global carbon dioxide emissions, accounting for approximately 7–8% of overall emissions [27]. The durability and productivity of cementitious substances are due to the abundance of calcium silicate hydrate particles in the cement [28]. These particles possess unique properties due to their nanoscale size, including strong binding capabilities. As a result, nanoparticles have gained prominence as concrete additives, improving their efficiency and performance [29,30]. The utilization of nanomaterials has emerged as a widely embraced strategy in the advancement of sophisticated cement composites [[31], [32], [33]]. Cement formulations enriched with nanomaterials elevate the materials mechanical attributes and introduce innovative properties. These attributes encompass diminished porosity, heightened resistance to frost, electrical conductivity, self-repairing capabilities, and self-cleaning functionalities [34,35].

**Fig. 1:**
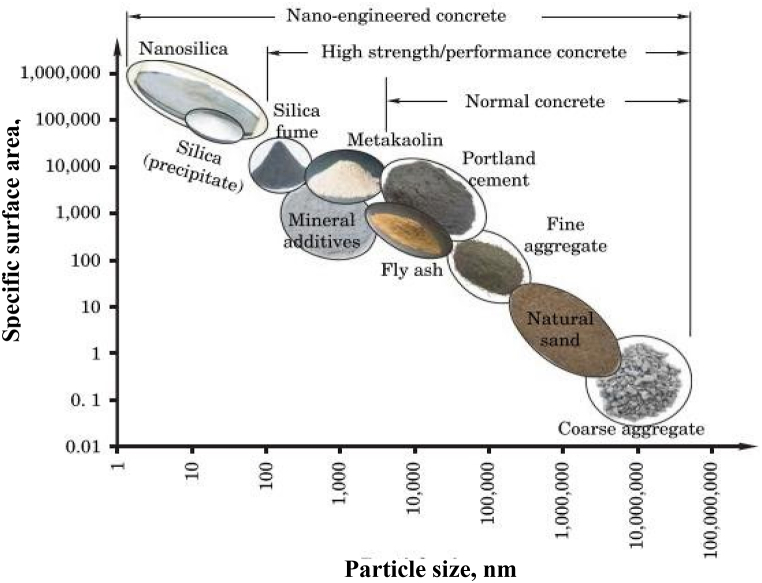
Particle size distribution and specific surface area of concrete constituents [36].

Due to their minute size, nanoparticles exhibit an exceptionally vast specific surface area (Fig. 1). The properties of cementitious materials can be significantly altered by adding these nanoparticles, typically in amounts as low as a few percent. Various nanoparticles have been studied for their potential to enhance cementitious composites. For instance, silica nanoparticles enhance the pozzolanic reaction and refine the cement matrix [37], carbon nanotubes improve tensile strength and electrical conductivity [38], and graphene oxide increases toughness and barrier properties [39]. Among these, nano-titanium dioxide (nano-TiO_2_) is distinguished by its unique photocatalytic properties, which enhance durability and offer additional benefits such as self-cleaning and air-purifying capabilities [40]. These multifunctional properties make nano-TiO_2_ a promising candidate for sustainable construction practices. Unlike other nanoparticles, nano-TiO_2_ provides enhanced durability and environmental benefits, setting it apart in the field of construction materials. Notably, titanium nano-oxides have demonstrated their ability to enhance resistance, expediting hydration processes, and imparting self-cleaning properties to the material [[41], [42], [43]]. Photocatalysis in cement-based substances has been explored since the beginning of the 1970s, with Akira Fujishima initiating the field. Fujishima's inquiry focused on titanium dioxide's photocatalytic and superhydrophilic features, which resulted in the discovery of the Honda-Fujishima effect. Since then, photocatalysis has been an ongoing area of research, with steady inclusion in cement-based outcomes. Because titania has photocatalytic capabilities, modern scientific study has concentrated more on adding nano titanium dioxide (TiO_2_) to concrete compositions. TiO_2_ is a microorganism that can give cement-like materials beneficial properties like self-cleaning and air-purifying [44]. The revolutionary work of Akira Fujishima, photocatalysis in cement-based materials has been studied since the early 1970s. The Honda-Fujishima effect was discovered due to his study on titanium dioxide's photocatalytic and superhydrophilic characteristics. This finding has sparked more investigation and the progressive incorporation of TiO_2_ into cement-based products [45]. The possible uses of titanium dioxide in concrete composites and other building materials are attracting the attention of scientists, engineers, and researchers. TiO_2_'s distinctive characteristics work well with standard building materials [9]. TiO_2_ is an inactive nano-filler that can change pore architecture and clog pores despite being generally inert. Because of its small size, it can act as a nucleation site in the cement matrix at the nanoscale, increasing stiffness in the early stages and durability over time [[46], [47], [48], [49]]. The impact of TiO_2_ on the longevity, flexibility, and durability of cement concrete and mortar is a potential field of study. By utilizing titanium dioxide's many attributes, especially its photocatalytic and fortifying qualities, the current investigation seeks to improve the structural integrity of the building materials [50]. TiO_2_ is esteemed for its extraordinary qualities, and adding it to materials that resemble cement could greatly increase its functionality. Perhaps the most remarkable property of TiO_2_ is its capacity for photocatalysis, which can reduce the growth of microorganisms on concrete surfaces and improve the general endurance of these constructions. Furthermore, by lowering the need for harsh chemical treatments, TiO_2_ promotes environmentally conscious building practices while extending the lifespan and durability of structures. The wide-ranging research looks into the impact of introducing nano TiO_2_ (titanium dioxide) into mixtures for concrete. It assesses the positive aspects of TiO_2_, such as its influence on physical properties, longevity, and microstructure, along with the ability to revolutionize the field of building. The conversation discusses contemporary issues, emphasizing nanomaterial's vital function in improving product qualities, refining efficiency, and promoting lasting viability in the building industry.

## 2 TiO_2_ fundamentals

Titanium dioxide (TiO_2_) nanoparticles are generated using handles and production steps, resulting in numerous particles varying across sizes from 1 to 100 nm. Their nanometer dimension provides a substantial specific area of surface, substantially raising their ability to react [51]. Titanium dioxide nanoparticles became known as an important nanomaterial in the field of composites made of cement due to their unique characteristics and diverse uses [52]. Nano-titanium dioxide (NT), often generated in spherical or ellipsoidal shapes, has become popular in cement-based buildings. This nanomaterial made from titanium dioxide, has properties that make it extremely adaptable and ideal for a wide range of applications in the industry [[53], [54], [55], [56]]. Titanium dioxide is widely used for various reasons, including its great chemical properties, low toxicity, affordability, anti-corrosion electrical aspects, and outstanding photocatalytic activities [7,57,58].

**Fig. 2:**
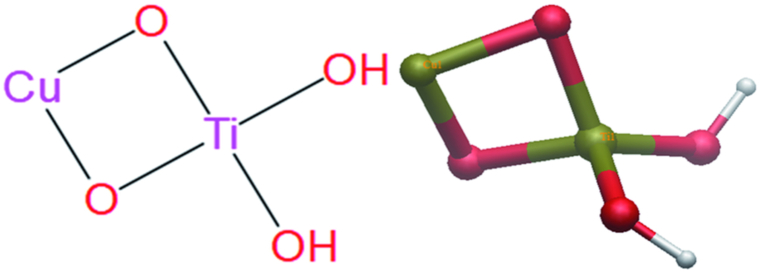
The TiO_2_Crystal structure.

Fig. 2 depicts the analysis of the crystalline structure of titanium dioxide (TiO_2_) in 2D and 3D models. Three distinct stages of TiO_2_ are possible: rutile, anatase, and brookite [59]. These stages have particular qualities that make them suitable for various prospective applications [60,61]. TiO_2_ has photocatalytic attributes and is a nanoscale semiconductor, creating multiple possible applications in several industries. The two most frequent crystalline forms of titanium dioxide are anatase and rutile [62]. Rutile finds extensive application as a pigment in the paper, textile, paint, and plastics industries. Because of its unique photocatalytic abilities, anatase is frequently employed in air and water purification systems [63]. It is significant that the third structure, brookite, causes industrial hurdles and that its entire potential usage spectrum is still largely undiscovered [64,65]. Of the two common types, anatase is the better option for a range of elemental coatings because it has more photocatalytic activity than rutile. Anatase has proven remarkably efficient at breaking down organic and inorganic impurities. Further studies [66,67]have demonstrated that the complementary combination of the anatase and rutile phases greatly increases photocatalytic activity, which is compatible with conclusions from additional research. The combination of anatase and rutile offers fascinating prospects for environmental remediation and several other sectors. The wide range of possible uses of titanium dioxide (TiO_2_) has led to much study on the material. Among the applications for which it has been researched are as a white pigment, in hydrolysis [68], in generating electricity [69], and as an addition to various building materials, including cement, concrete, tiles, and windows. Indeed, these applications make use of the exceptional properties of TiO_2_, which include its sterilization, deodorization, and antifouling capabilities [[70], [71], [72], [73], [74]].

**Fig. 3:**
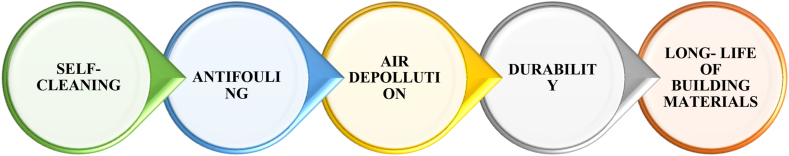
Functionalized titanium dioxide nanomaterials in the construction sector.

When TiO_2_ is integrated into concrete, it functions as a photocatalyst when exposed to light, initiating chemical reactions on its surface and the Functionalized Titanium Dioxide Nanomaterials in the Construction Sector is shown in Fig. 3. TiO_2_ nanoparticles are well-known for their photocatalytic activity, enabling them to degrade organic pollutants and improve the self-cleaning properties of construction materials. The self-cleaning properties of TiO_2_ have found practical applications in building and paving materials, exemplified by structures such as the Jubilee Church in Rome, Italy [75]. With a band gap energy of 3.2 eV and a matching wavelength of 385 nm, titanium dioxide (TiO_2_) has special features that allow it to form a pair of electrons and holes when subjected to ultraviolet radiation of this particular wavelength [76]. TiO_2_'s self-cleaning properties are based on this photo-induced process, which makes it an excellent addition to building aspects where maintenance and purity are essential. Due to TiO_2_'s photocatalytic qualities, dangerous airborne pollutants including nitrogen oxides (NOx) and volatile organic compounds (VOCs) can be reduced and decomposed, improving the air quality.

TiO_2_ is an essential part of concrete due to its significant light-scattering capabilities, ability to scatter ultraviolet (UV) rays and photocatalytic authority. TiO_2_ is a photocatalyst that may extensively alter and eliminate pollutants, especially organic materials, by producing pairs of electron-holes when exposed to light. Suppose organic compounds undergo exposure to ultraviolet (UV) light. In that case, the metal oxide TiO_2_ is particularly good at creating enthusiastic holes for electrons, which can accelerate water decay regardless of moderate settings. TiO_2_ is superior to other oxides of metal due to its photocatalytic feature, which makes it a great option for breaking down organic contaminants. Several investigations have also demonstrated that TiO_2_ has minimal toxicity and sunlight absorption [[77], [78], [79], [80], [81]].

## 3 The utilization of nano-titanium dioxide in materials based on cement

**Fig. 4:**
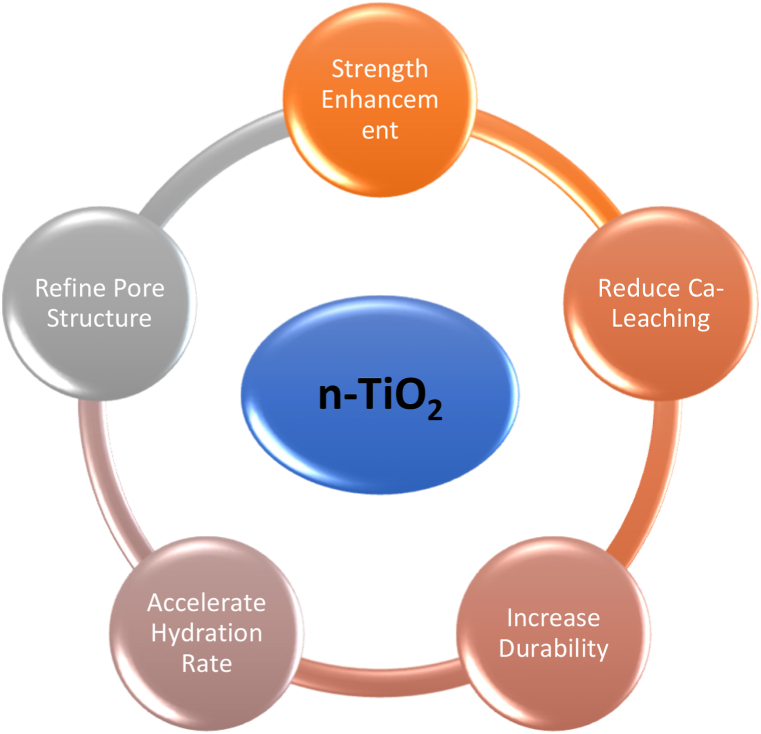
Behavior of Nano-TiO_2_ in the cement matrix and enhanced properties.

The application of nanomaterials, specifically nano-titanium dioxide (nano-TiO_2_), to the manufacturing of industrial concrete has attracted substantial attention in the last ten years. The combined use of nano-TiO_2_ greatly improves the properties and uses of cementitious materials. Due to its several advantages such as chemical rigidity, photocatalytic qualities, self-cleaning abilities, affordability, and adaptability which can be attributed to its small size, nano-TiO_2_ has been thoroughly investigated [[82], [83], [84]]. This nanomaterial significantly influences cement hydrolysis and the structure of calcium silicate hydrate (C-S-H) gel. The enhanced energy levels and specific surface area of nano-TiO_2_ reduce both macro- and micro-pores while raising the number of small pores in the cement matrix. These enhancements result in improved mechanical properties, durability, and general functionality of the cement-like materials, presenting nano-TiO_2_ as a vital component in modern building innovations. This refinement in pore structure generally enhances concrete strength, although an excess of nanopores may potentially diminish strength [85]. Incorporating TiO_2_ nanoparticles into concrete yields various advantageous effects, including enhanced flexural fatigue performance, increased abrasion resistance, improved homogeneity, greater compaction, reduced pore volume and size, and diminished permeability (Fig. 4). The acceleration of pozzolanic reactions by TiO_2_ nanoparticles results in heightened cement hydration rates, increased intensity of heat peaks, and reduced setting times, proportionate to the nano-TiO_2_ content. This reduction in setting time can prove cost-effective by saving construction time and expenses. Additionally, TiO_2_ nanoparticles mitigate water loss; and enhance hydrophilicity, ultimately reducing drying shrinkage in cementitious materials [86,87]. Notably, the inherent tendencies of nano-TiO2, such as its tiny particle size, increased surface energy, and strong intermolecular van der Waals interactions, make it susceptible to aggregation. Once agglomeration occurs, achieving a uniform dispersion of nano-TiO_2_ becomes challenging due to cohesive forces [88]. Beyond conventional construction materials, the integration of nano-TiO_2_ in materials made of cement has garnered significant focus for its potential to introduce novel functionalities. These functionalities include self-cleaning and antimicrobial properties, offering practical applications in mitigating urban air pollution by reducing concentrations of both organic and inorganic pollutants [89,90].

Titanium dioxide (TiO_2_) integrated into concrete imparts remarkable self-cleaning capabilities through photocatalytic processes, efficiently degrading contaminants from various sources. Due to its inherent stability and resistance to corrosion, TiO_2_ is produced in substantial quantities. When illuminated by sunlight, nano-TiO_2_ efficiently transforms organic pollutants and fragments outside of concrete into water and carbon dioxide, which can be readily eliminated by rainfall or simple washing [91]. Due to its larger surface region, the improved photocatalytic ability of titanium dioxide (TiO_2_) in its nanoparticle form imparts concrete with self-cleaning and sterilizing traits [92,93]. Integrating nano-titanium dioxide into building materials induces advantageous microstructure transformations, including lower pores and broadened porous responses. The elevated exterior region of TiO_2_ nanoparticles drastically promotes pozzolanic actions, consequently improving the concrete's asset. However, obstacles may complement these helps, such as raised water absorption and potential impacts on concrete workability. Despite these challenges, the advantages of integrating nano-TiO_2_ into cementitious materials are substantial, establishing the way for changes in the building process industry. Moreover, the special capacity of TiO_2_ nanoparticles to form connections with the gel C-S-H and each other enhances the overall influence of concrete [87]. Concrete incorporating TiO_2_ presents applications in pavements, potentially reducing tropospheric O_3_ levels and other beneficial properties [94]. This suggests a broader spectrum of applications for TiO_2_-enhanced concrete in addressing environmental and urban challenges. For cement-based composites to possess antimicrobial, self-purifying, and traits that reduce air pollution, efficient titanium nano-oxide dispersion is essential. However, titanium dioxide nanoparticles tend to aggregate within the cement matrix, negatively impacting their performance [[95], [96], [97]]. Various techniques, such as ultrasonic energy mixing, superplasticizer, and water premixing, shear mixing with concurrent component mixing, and shear blending with previous nanoparticles premixing with water and superplasticizer, are employed to disperse these nanoparticles [98]. Despite these efforts, complete prevention of accumulation is not always feasible due to factors like environmental pH and salt present in the pore solution. Studies indicate that even well-distributed titania nanoparticles in water tend to re-agglomerate when added to the cement matrix. A polycarboxylate superplasticizer causes contact between the strongly charged nano-titania surface and the calcium ions in the cement mixture, leading to re-agglomeration [95]. Consequently, one of the significant challenges for the future lies in discovering efficient methods to disperse nano-additives in cement. Cement hydration relies on titanium nano-oxide effectively dispersed throughout the cement matrix [99]. The integration of titania at the nanoscale within cement exhibits a discernible augmentation in the cement hydration process, with a pronounced impact observed, particularly during the initial phases. The nano-sized particles are postulated to function as sites conducive to heterogeneous nucleation, thereby expediting the genesis of hydrated cement and conceivably exerting a discernible influence on the inherent properties of the resultant concrete [90]. Moreover, nano-titania imparts supplementary nucleation sites for the crystalline calcium-silicate-hydrate (C-S-H) phase, concurrently mitigating the formation of calcium hydroxide (CH) hydrate. This dual effect culminates in a cement matrix characterized by heightened compactness and reduced porosity, thereby enhancing the robustness and longevity of cementitious composites [43,90,100].

## 4 Fabrication of nano-titanium dioxide cementitious composites

**Fig. 5:**
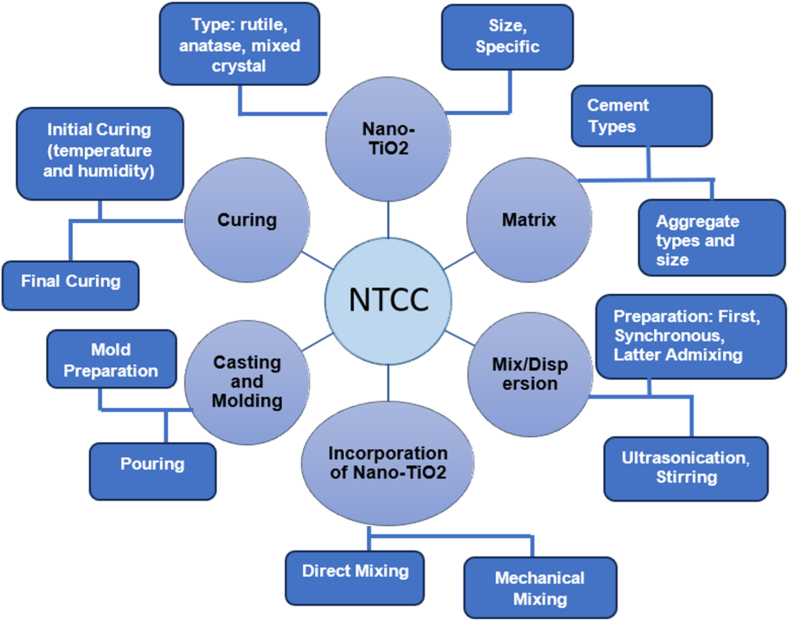
Schematic Illustration of production methods for nano titanium dioxide/cementitious composites (NTCC).

The production of cementitious composites incorporating Nano-TiO_2_ encompasses several steps, such as material selection, mixing/dispersing, molding, and curing. Choosing nano-TiO_2_, which includes its type, size, and surface area, is vital in establishing the overall achievement of the mixture material at the microscopic stages. Effective combining and distribution for nano-TiO_2_ (as revealed in Fig. 5) are important for accomplishing consistency and the envisioned characteristics in the composite material. Still, this threshold deal is demanding owing to the inadequate dimension of nano-TiO_2_ particles, their higher surface energy, and their respective positions inclination to agglomerate.

Its particle size notably influences the physical characteristics of TiO_2_. Table 1, Table 2 presents the physical properties and mix proportions of TiO_2_ from various studies.

**Table 1:** Physical properties of TiO_2_.

Type	Purity/Structure Name	Density (g/cm^3^)	Specific Surface area (m^2^/g)	Average particle size (nm)	Reference
Powder	Anatase	3.9	–	15–30	[60]
	Anatase/99.8 %	0.05	260	15	[101]
	99.7 %/Anatase	40–60	240 ± 50	15	[102]
	Rutile/≥99 %	4.6	40	20	[103]
	>99 %	2.61	150 ± 20	10–20	[104]
	99.9 %	<0.13	155 ± 12	15 ± 3	[105]
	Anatase/99 %	–	58.8	21	[43]
	Rutile/>99.9 %	3.74	163	10–20	[106]
	Anatase/99 %	<0.15	153 ± 10	15 ± 2	[86]
	Anatase/99.8 %	3.16	50–100	20	[107]
	>99.9 %	<0.15	165–17	20 ± 5	[108]
	Anatase/97 %	–	45–55	20–30	[109]
	>99.9 %	<0.24	48 ± 10	18 ± 3	[110]
	Anatase/>97 %	–	45–55	20–30	[90]
	Anatase/99.9 %	4	150	10	[111]
	Anatase/99.9 %	3.9	240	15	[18]

**Table 2:** Mix proportions in Nano-TiO_2_ cementitious composites.

Matrix types	Cement types	Aggregate types and size	Content of Nano-TiO_2_ (wt. % of cement)	Admixture types and content	Admixture solid content	Proportion of C: W:S	References
Concrete	Portland cement (O·P·C 42.5R)	Quartz sand (0.12–0.83 mm)	0.78 vol%	Polycarboxylate Superplasticizer	44 %	1:0.375:1.375	[111]
			2.32 vol%				
			3.88 vol%				
	Ordinary Portland cement (OPC)	crushed natural gravel (12 mm)	0,1,2,3,4,5	HRW polycarboxylate	40	C:S: 1:1.80	[103]
		natural sand (0.6 mm)					
	Ordinary Portland Cement	Fine natural river sand	2	Superplasticizer (Glenium 51P)	34%–36 %	1:0.48: 0.51	[101]
		Crushed stone (5–12 mm)					
	Portland cement (OPC 42.5R)	Quartz sand (0.12–0.83 mm)	1, 3, 5	Superplasticizer	–	1:0.375:1.375	[112]
	Type II Portland Cement	Sand (4.75 mm)	1, 2, 3,4, 5	Polycarboxylic-ether type Superplasticizer	–	1:0.38: 2.23	[108]
		Coarse aggregate (12.5 mm)					
	53 Grade Ordinary Portland Cement	Fine aggregate	2, 3, 4, 5, 6	Superplasticizer	40 %	–	[113]
		Coarse aggregate (12.5 mm)					
	ordinary Portland cement (CEM I 42,5R)	Sand (4.0 mm)	1,2, 3	Polycarboxylic-ether polymer-type Superplasticizer	–	1:0.3:0.35	[114]
		Coarse aggregate (8.0 mm)					
	OPC Ultra Tech Cement 53 Grad	Coarse aggregate (20 nm −10mm)	0.5 %	Superplasticizer	0.8 %	1:1.62: 3.39	[60]
		River Sand	1.0 %				
			1.5 %				
	ordinary Portland cement CEM I 42.5	Basalt	1,3,5	Polycarboxylate Superplasticizer	25 %	–	[104]

## 5 Impact of nano-titanium dioxide on various characteristics of cementitious materials

Many studies have been conducted on combining nano-titanium dioxide (nano-TiO_2_) with concrete composites because it can drastically affect their fundamental attributes. The outcome of nano-titanium dioxide in materials composed of cement is contingent upon aspects involving its quantity, the sort of cement utilized, and the surrounding circumstances. These variables influence the substances fresh properties, mechanical characteristics, durability, and microstructure.

### 5.1 Effect on fresh properties

Adding nano-TiO_2_ to cement composites may impart fresh characteristics such as workability, consistency, and setting time while altering the texture of substances via size dispersion shifts. The smaller sizes of those nanoparticles may have a major effect on these properties, improving or decreasing workability according to factors such as dosage and size of the particle. The microscopic particles must be meticulously diffused and the resulting solution must be calibrated proportionately to achieve fresh features. Likewise, the distribution of particle sizes of the composite constituents could substantially affect mortar's fresh qualities, with nanomaterials capable of producing noticeable alterations due to their small size.

#### 5.1.1 Workability

**Fig. 6:**
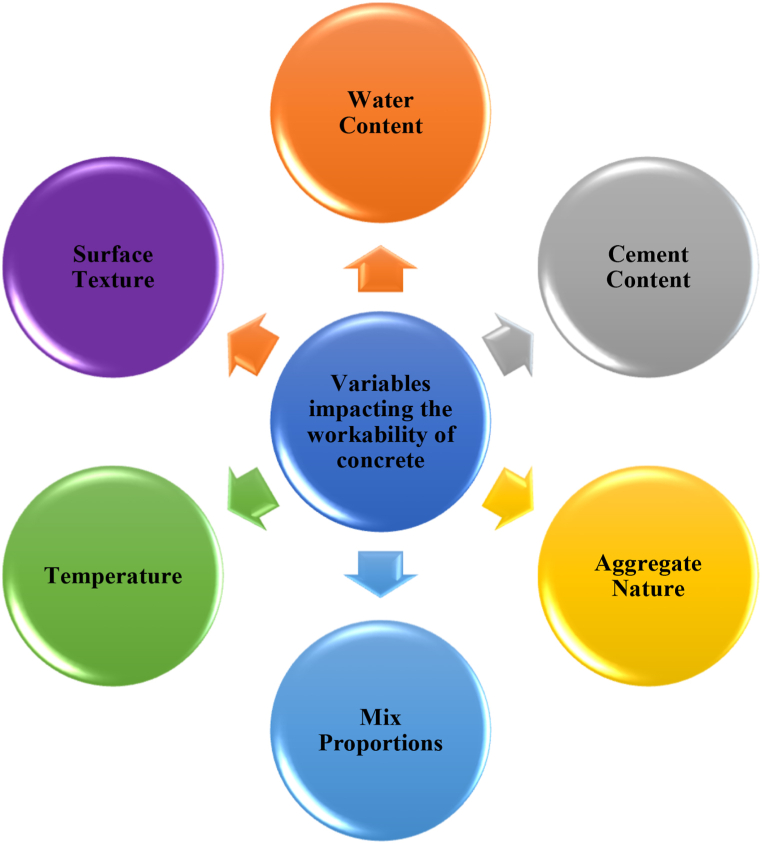
Influential factors on the workability of concrete.

The level of significance of nano-TiO_2_ aggregation influences the workability of cementitious composites, which includes factors like slump and slump flow [99] and the Influential Factors on the Workability of Concrete are shown in Fig. 6. Gopalakrishnan et al. [86], studied the detrimental effect of nano-TiO_2_ on mortar workability. Mortar containing 8 %–10 % nano-TiO_2_ and 15.2 %–15.5 % water content demonstrated excellent workability. Zhang et al. [91], augmenting the amount of nano-TiO_2_ in cementitious composites resulted in a reduction of slump. In the case of a W/C (water-to-cement) ratio of 0.6, slumps diminished by 2.8 %, 19.8 %, and 20.8 % with 1, 3, and 5 percent of nano-TiO_2_, respectively. Slump flow also decreased by similar percentages for the same nano-TiO_2_ concentrations. Joshaghani et al. [110] investigated the influence of 3.0 and 5.0 wt percent nano-TiO_2_ on the fresh properties of self-compacting concrete. Workability slightly increased with 3.0 wt% nano-TiO_2_ but decreased with 5.0 wt% due to higher water demand caused by increased nano-TiO_2_ content. The findings from slump flow tests indicated a diminished diameter attributed to the expansive surface area of the nanoparticles and their propensity for water absorption. Variations were observed in the L-box and V-funnel results, where a 3 % nanoparticle inclusion exhibited enhanced workability, while a 5 % inclusion resulted in a decrease.

Mohseni et al. [115] reported that the impact of increasing nano-TiO_2_ content, and the slump flow diameter of cementitious composites also increased. Specifically, the slump flow diameter measured 258 mm upon incorporating 5 wt % of NT (nano-titanium), signifying a 5.3 % augmentation compared to cementitious composites to control. However, the V-funnel flow time for the cement-based composites increased as the NT content increased, potentially attributable to the relatively elevated water-cement ratio (0.53), which could constrain NT's mitigation capacity. Salemi et al. [101] noted a decline in the workability of a concrete blend with the introduction of 2 % NT. Specifically, the control mixture had a slump of 120 mm, while the NT mixture had a slump of 60 mm. The results indicate that adding 2 % NT to the concrete mix significantly decreased workability. Meng et al. [116] directed their attention to the flow characteristics of mortar mixtures modified with nano-titanium (NT). Various levels of cement replacement with NT were explored, namely 0 %, 5 %, and 10 % by weight, while maintaining a constant water-to-binder (w/b) ratio of 0.5 %. The outcomes indicated a decline in the fluidity of the mortar mixtures with an increase in NT content; notably, the fluidity diminished by 21 % with the incorporation of 5 % NT, and incorporating 10 % nano-TiO_2_ led to a 40 % reduction in fluidity. Nano-TiO_2_ within the mortar mixture decreased its fluidity, potentially affecting the materials workability and handling.

#### 5.1.2 Setting time

The presence of nano-TiO_2_ can have an enormous effect on the setting time of composite materials, potentially accelerating or delaying the procedure. The outcomes are determined by nano-TiO_2_ quality and cementitious mixture composition. The setting time is calculated by assessing the mortar resistance to piercing till it strikes the 3.5 MPa threshold, which indicates the initial phase of concrete setting up. R. Gopalakrishnan et al. [86]incorporating nano-titanium dioxide (NT) into concrete paste significantly reduced initial and final setting periods. As NT ratios ascended from 0 % to 10 %, the decreases in setup times appeared more noticeable. Zhang et al. [91]revealed that integrating 1 %, 3 %, and 5 % nano-TiO_2_ resulted in an incremental reduction in cement preliminary setting durations of 37.9 %, 63.4 %, and 76.5 %, respectively, coinciding with increased NT percentage. Similarly, the final setting times declined 15.7 %, 37.4 %, and 46.2 %, respectively, upon adding 1 %, 3 %, and 5 % nano-TiO_2_ content. Ma et al. [117] documented that the inclusion of 3 % nano-titanium dioxide led to reductions in initial and final setting times as contrasted with pure cement. Janus et al. [118] noted a notable decrease in initial and final setting times in concrete containing 5 % nano-titanium dioxide as opposed to unmodified concrete. Lee et al. [90] have observed notable reductions in initial and final setting durations in concrete formulations incorporating concentrations of 5 and 10 wt% of nano-titanium dioxide. This observation indicates that greater concentrations of nano-TiO_2_ expedited the setting process in the concrete matrix. Daniyal et al. [119]exhibited that the provision of nano-titanium dioxide appears to be an acceleration, competently minimizing the setting time in cement-based composite materials. The result signifies that the existence of NT might speed up the setting process of these components. Comparably in a distinct investigation, Wang et al. [87]assessed the setting time of cement mixes comprising 1.0 wt% to 5.0 wt% nano-TiO_2_ according to distinctive curing conditions. These outcomes disclosed that the two more substantial curing temperatures and raised nano-TiO_2_ materials decreased the setting time.

### 5.2 Effect on mechanical characteristics

Including nano-TiO_2_ greatly improves the mechanical qualities of concrete, which is crucial for assessing the material's strength and longevity. Calcium silicate hydrate (C-S-H) gel is formed with the help of nano-TiO_2_, which functions as a nucleation site to diminish pore appearance and increase material rigidity. This enhancement is most effective within specified concentration limits, as validated by pertinent research [85]. Variables such as concrete mix formulation, curing methodologies, and the utilization of additives exert discernible influences on the mechanical characteristics of concrete. Nano-TiO_2_ significantly contributes to a marked escalation in compressive and flexural strength within cement-based materials by serving as filler and optimizing particle packing.

#### 5.2.1 Compressive strength

**Fig. 7:**
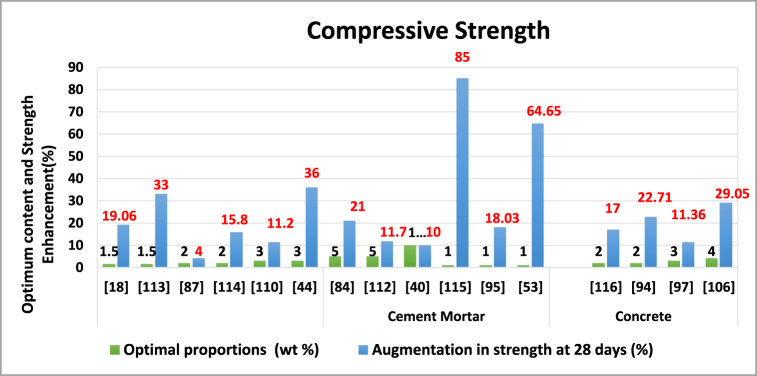
Optimal Nano-TiO_2_ contents for enhancing compressive strength.

Nanoparticles, like nano-TiO_2,_ enhance concrete compressive strength by serving as nuclei for cement phases, promoting hydration, densifying the microstructure, reducing porosity, and filling pores [120,121]. However, when using a high nano-particle content, adjustments to the water superplasticizer dosage are necessary to prevent accumulation and mitigate self-desiccation and cracking. High nanoparticle content can also lead to challenges in achieving uniform dispersion, increasing weak zones that can reduce concrete strength. Table 3 and Fig. 7 encapsulate the influence of Nano-TiO_2_ on the compressive strength of composite materials incorporating cement.

**Table 3:** The influence of Nano-TiO_2_ on the compressive strength of composite materials.

Matrix category	Size (nm)	W/C	Proportions (wt. %)	Optimal proportions (wt. %)	Augmentation in strength at 28 days (%)	Reference
Cement Mortar	15	0.35	0.5,1,1.5	1.5	19.06	[18]
	10–30	0.45	0.5, 1, 1.5, 3	1.5	33	[122]
	15	0.5	1,2,3,4,5	2	4	[94]
	21	0.35	1, 2, 3,	2	15.8	[123]
	21	0.5	1, 2, 3,	3	11.2	[117]
	15	0.485	1,3,5	3	36	[51]
	25	0.4	1, 3,5	5	21	[91]
	30	0.45	1,3,5	5	11.7	[119]
	21	0.485	5,10	10	10	[43]
Concrete	10–30	0.5	0.5,0.75, 1, 1.25, 1.5	1	85	[124]
	15	0.42	1, 3, 5	1	18.03	[102]
	15–30	0.33	0.5,1,1.5	1	64.65	[60]
	25 ± 5	0.30	1,2,3	2	17	[125]
	15	0.48	2	2	22.71	[101]
	10	0.4	1,3,5	3	11.36	[104]
	10–20	–	2,3,4,5,6	4	29.05	[113]

#### 5.2.2 Split Tensile strength

**Fig. 8:**
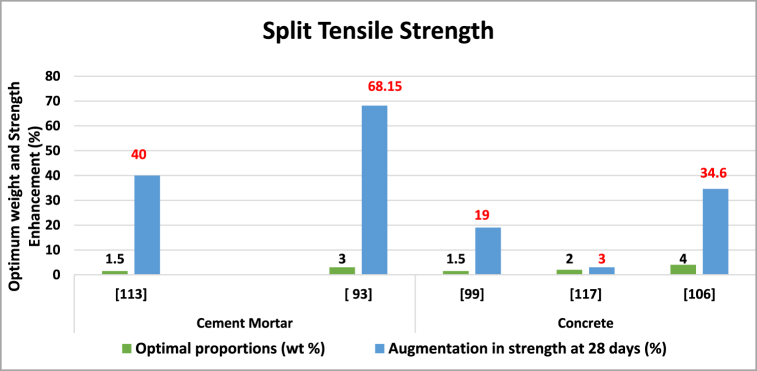
Optimal Nano-TiO_2_ contents for enhancing Split Tensile strength.

Nano-TiO_2_ enhances compressive strength more than tensile strength in cementitious composites. It accelerates hydration, improves particle packing, and reduces larger pores, enhancing overall performance and microstructure. However, dispersion defects can lead to weak zones [112]. The impact of Nano-TiO_2_ on the tensile strength of composite materials with cement-like properties has been consolidated in Table 4 and Fig. 8.

**Table 4:** The influence of Nano-TiO_2_ on the (split) tensile strength of composite materials.

Matrix category	Size (nm)	W/C	Proportions (wt. %)	Optimal proportions (wt. %)	Augmentation in strength at 28 days (%)	Reference
Cement mortar	10–30	0.45	0.5, 1, 1.5, 3	1.5	40	[122]
	–	0.58	1,2,3,4,5	3	68.15	[100]
Concrete	10–20	0.52	0.5,1,1.5,2,2.5,3	1.5	19	[106]
	15	0.40	1,2,3,4	2	3.0	[126]
	10–20	–	2,3,4,5,6	4	34.60	[113]

#### 5.2.3 Flexural strength

**Fig. 9:**
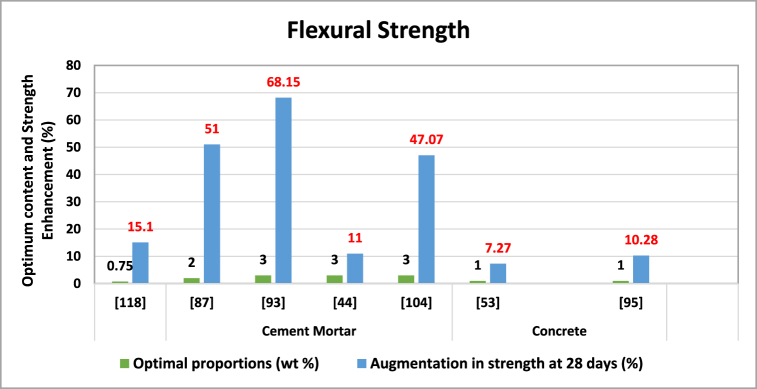
Optimal Nano-TiO_2_ contents for enhancing Flexural strength.

A synergistic application of careful mix design, appropriate additive inclusion, and material selection is necessary to support the flexural strength of cement-based composites using TiO_2_, comparable to improving compressive strength. This enhancement increases the concrete's resistance to bending and cracking, signifying improved cement toughness [106]. Table 5 and Fig. 9 summarize the impact of Nano-TiO_2_ on the flexural strength of composite materials incorporating cement.

**Table 5:** The influence of Nano-TiO2 on the flexural strength of composite materials.

Matrix category	Size (nm)	W/C	Proportions (wt. %)	Optimal proportions (wt. %)	Augmentation in strength at 28 days (%)	Reference
Cement Mortar	15	0.5	0.25, 0.75, 1.25, 1.75	0.75	15.1	[127]
	15	0.5	1,2,3,4,5	2	51	[94]
	–	0.58	1,2,3,4,5	3	68.15	[100]
	15	0.485	1,3,5	3	11	[51]
	10	0.375	1,3,5	3	47.07	[111]
Concrete	15–30	0.33	0.5, 1,1.5	1	7.27	[60]
	15	0.42	1, 3, 5	1	10.28	[102]

### 5.3 Durability

Nano-TiO_2_ significantly enhances the durability of cementitious composites through several mechanisms. It reduces water permeability, reinforces resistance against chemical attacks, bolsters resilience to environmental stressors like UV radiation, and diminishes porosity within materials based on cement. These impacts result in a more compact and homogeneous microstructure with smaller pore sizes, resulting in lower permeability and increased overall durability [110,115,128,129]. Utilizing Nano-TiO_2_ in cement-based composites is associated with improvements across diverse durability indicators, including permeability, electrical resistivity, resistance to carbonation, freeze-thaw resilience, ultrasonic pulse velocity, and sulfate attack. However, it's worth noting that an excessively high content of nano-titanium dioxide could potentially exert a negative impact on durability [114,130]. Ensuring the long-term durability of cement composites primarily depends on factors such as the compactness of the cement paste and the nature of the cement binder, particularly concerning the existence of calcium hydroxide and ettringite as well as the boundary region connecting the cement paste with the aggregate [27,131].

#### 5.3.1 Permeability

The impact of permeability on the longevity and functionality of concrete and cementitious materials is well-documented in the extensive research literature [102,132,133]. Permeability plays a pivotal role in determining the operational lifespan of structures made of concrete, thereby influencing their comprehensive life-cycle expenditures. To mitigate permeability challenges in creating cement-based products, nano-additives such as nano-titanium dioxide emerge as a compelling and productive strategy. These nanoparticles enhance the materials microstructure while simultaneously reducing porosity, resulting in a significant reduction in permeability [86,110,134]. Analyses constantly indicate that a decline in permeability is achieved at an appropriate level when the proportion of nano-TiO_2_ in composites made from cement is doubled. The result lowers permeability and optimizes mechanical characteristics at the same time. Assessing the water retention of concrete composites serves a purpose since it offers substantial data on the porous nature of the component [26]. By drastically reducing water absorption, nano-TiO_2_ promotes physical strength and reduces humidity-related challenges. Concrete construction must minimize chloride ion permeability in caustic situations. It has been demonstrated that Nano-TiO_2_ is very efficient in reducing the reach of chloride ions, improving its ability to resist erosion caused by chloride.

**Fig. 10:**
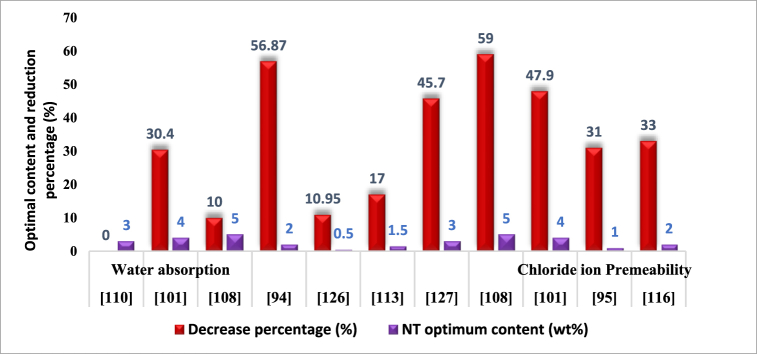
The ideal concentrations of nano-TiO_2_ for permeability reduction in cement-based composites associated with reduction percentages.

Chloride can harm the protective layer on reinforced steel. When that happens, it might cause the steel to rust, which can lead to serious problems like structural failure. Nano-TiO_2_ helps improve the microstructure of cementitious materials, which restricts chloride and water ions from moving through the material. This is responsible for its effectiveness in keeping dangerous chemicals from penetrating the material. In general, adding Nano-TiO_2_ with cement paste together greatly improved the durability of cementitious materials considering factors such as permeability and improved microstructure which can strengthen its resistance against moisture content and domineering ions, that involve chloride [116]. Table 6 and Fig. 10 provide insights into the influence of Nano-TiO_2_ (NT) on various permeability parameters in concrete and mortar. It also highlights the optimal NT usage percentages in mixtures to reduce these characteristics and the resulting reduction percentages.

**Table 6:** The optimal Nano-TiO_2_ concentrations in cementitious materials for the permeability reduction and the corresponding percentage reductions.

Decrease percentage (%)		Matrix type	Investigated contents (wt.%)	NT optimum content (wt.%)	Curing time (Day)	Reference
Water absorption	40–65	Cement Mortar	1,2,3,4,5	3	28	[117]
	30.4	Concrete	1,2,3,4,5	4	7	[108]
	10	self-compacting mortar	1,3,5	5	90	[115]
	56.87	concrete	2	2	28	[101]
	10.95	Concrete	0.5,1,1.5,2	0.5	90	[135]
	17	Cement Mortar	0.5,1,1.5,3	1.5	28	[122]
	45.7	Concrete	1,2,3,4	3	7	[136]
Water vapor permeability coefficient	43.9	Cement Mortar	1,2,3,4,5	3	28	[100]
Water absorption coefficient	40	Cement Mortar	1,2,3,4,5	3	28	[100]
Chloride ion Permeability	59	self-compacting mortar	1,3,5	5	90	[115]
	47.9	Concrete	1,2,3,4,5	4	90	[108]
	31	Concrete	1,3,5	1	28	[102]
	33	Concrete	1,2,3	2	28	[125]
Capillary water absorption	20.7	concrete	1,2,3,4,5	4	7	[108]
Gas permeability Coefficient	81	Concrete	1,2,3,4,5	4	28	[103]
	4	Mortar	1,2,3	2	28	[123]

#### 5.3.2 Carbonation

**Fig. 11:**
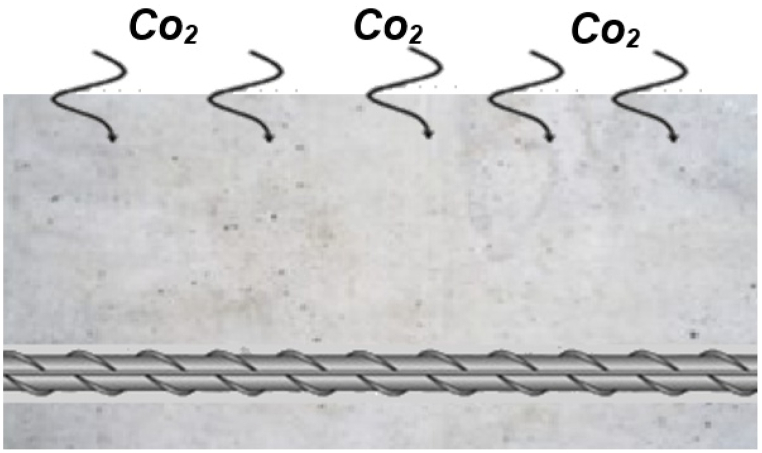
The process of concrete structure carbonation.

Carbonation is indeed recognized as the primary cause of corrosion in cement-based materials (Fig. 11). Atmospheric CO_2_ penetrates these materials, leading to structural deterioration [103,137]. In a comprehensive investigation by Moro et al. [138], the influence of CO_2_ absorption and carbonation in Nano-TiO_2_ (NT) altered cementitious paste was meticulously examined. Their results revealed a noticeable trend: the CO_2_ absorption of the adapted cementitious paste initially increased with rising NT content before later decreasing. Several contributing factors influenced this observed pattern. Firstly, introducing NT reduced the shape of calcium hydroxide (CH) crystals, thereby augmenting CH's reactivity with Carbon dioxide. Secondly, including NT resulted in a denser cementitious composite, impeding deeper areas of latent carbonation. Also having a higher cement-to-water ratio boosts the mix's ability to absorb CO_2_. More water in that ratio increases the pastes porosity and ability to absorb CO_2_. Ramachandran et al. [137]examined the carbonation depth in concrete with 40 wt% fly ash. Two nanomaterials were used in the study: a combination of one weight percent NT and one weight percent nano-CaCO_3_ and two weight percent nano-TiO_2_ (NT). Three environmental conditions were applied to concrete specimens: seawater immersion, increased carbonation, and standard atmosphere. The outcomes showed that adding nano-TiO_2_ alone decreased the concrete's resistance to carbonation. However, the carbonation resistance of the concrete increased significantly when NT and nano-CaCO_3_ were combined; in fact, the combined action of both nanomaterials proved to be more effective than that of each one alone. The influence of nano-TiO_2_ on the carbonation depth in self-compacting mortars containing 30 % fly ash by weight was investigated by Rao et al. [139]. Their results demonstrated that the binder-to-sand ratio affected how much NT affected carbonation resistance. Like the reference mortar, NT showed no carbonation depth at a binder-to-sand ratio of 1:1. Nevertheless, when NT concentration was above 0.5 wt percent, it negatively impacted carbonation resistance at a 1:2 binder-to-sand ratio. The capacity of nano-TiO_2_ to improve CO_2_ absorption in cementitious composites may be advantageous for unreinforced materials as it helps lower ambient CO_2_ levels, although carbonation can destroy reinforcing components in concrete. According to Shaaban et al. [140], nano-TiO_2_ positively affected mortars ability to withstand carbonation. Under their findings, the carbonation depth of mortars confronted with CO_2_ for 28, 56, and 90 days became considerable when NT was applied; the largest reductions were observed at 56 and 90 days. The filler effect of NT was shown to be responsible for the increased carbonation resistance by promoting the denser microstructure and lowering CO_2_ adsorption. As a result, a notable reduction in carbonation depth was noted over time, mostly due to the microstructure's compression brought on by NT. Further research by Zhang et al. [141] revealed a distinct pattern in the relationship between the carbonation depth of concrete and NT content, whereby an increase in NT content is consistently accompanied by a decrease in carbonation depth that eventually reaches minimal. The study noted that the concrete minimum carbonation depth occurred at an NT content of 3 wt%. In a study by Duan et al. [142], the influence of Nano-TiO_2_ (NT) on the depth of carbonation cementitious composites was demonstrated over 180 days. The findings suggested that compared to the reference materials, the carbonation depth for various NT contents was as follows: 1 % NT resulted in a 77 % reduction, 3 % NT led to a 62 % reduction, and 5 % NT showed a 42 % reduction.

#### 5.3.3 Freeze and thaw resistance

**Fig. 12:**
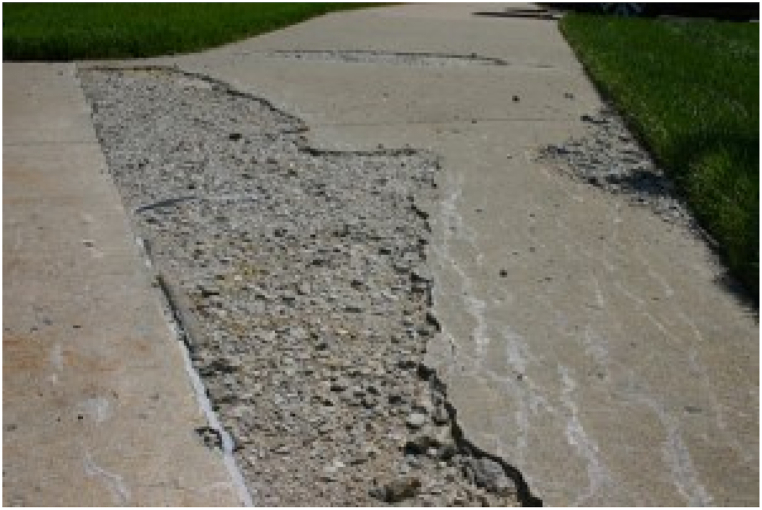
Impact of freeze-thaw cycles on concrete.

Durability in cold climates, especially frost resistance, is critical [104]. However, there is limited extensive research on the influence of Nano-TiO_2_ on the freeze and thaw resistance of cementitious materials (Fig. 12). Chunping et al. [129]explored the resistance to freeze-thaw cycling of high-performance cementitious material that included nano-TiO_2_(NT), both in the presence and absence of applied flexural load. The concrete specimens experienced 800 freeze-thaw cycles, and the results showed that adding Nano-TiO_2_ did not result in noteworthy changes concerning loss of material and the dynamic modulus ratio. Remarkably, a slight reduction in mass loss was noted in specimens subjected to bending stress when NT was applied. Salemi et al. [101]conducted a study to investigate the impact of incorporating nano-titanium dioxide (NT) on the resistance of concrete to frost. Also, conventional concrete was compared with concrete containing 2.0 wt% NT, subjecting to 300 freeze and thaw cycles. The results revealed a substantial positive influence of Nano-TiO_2_ on the frost resistance of cementitious materials. Following 300 freeze and thaw cycles, the regular concrete exhibited significant deterioration, including a 100 % strength reduction, an 84 % mass loss, a 28 % lesser length, and a 117 % higher water absorption. In contrast, the NT-containing concrete demonstrated markedly improved performance, experiencing only an 11.5 % strength reduction, a 5 % mass loss, a 2 % reduction in length, and a 20 % rise in water absorption. The improvement in frost resistance was linked to the forming of a more compact microstructure in the concrete containing NT. This denser microstructure resulted in lower water absorption, which, in turn, mitigated the detrimental impacts of freeze and cycles of thaw on the concrete.

#### 5.3.4 Sulfate attack resistance

**Fig. 13:**
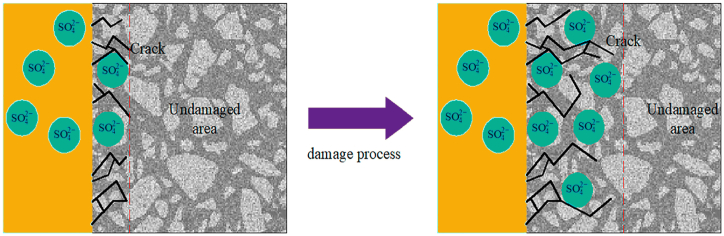
Formation resulting from sulfate attack.

Sulfate erosion poses a significant challenge to cementitious materials, causing weakening, expansion, fissures, and disintegration. Sulfate ions infiltrate these materials from groundwater, seawater, soil, and sewer pipes through diffusion and capillary action, initiating complex chemical reactions that form calcium aluminate and gypsum. This leads to the generation of internal pressure due to crystal growth, resulting in concrete swelling and damage (Fig. 13). The integration of NT enhances the durability of concrete by refining internal pore structures and reducing gypsum formation, thus reducing susceptibility to sulfate attack [143,144]. Additionally, nano-TiO_2_ enhances sulfate attack resistance in cement-based materials by creating a denser microstructure and reducing ionic transport [119]. Xu et al. [130]studied the influence of sulfate exposure on conventional cementitious materials and concrete enhanced with nano-titania. The findings indicated that nano-titania (NT) improved the concrete's ability to resist sulfate-induced damage while also decreasing both mass loss and the loss of compressive strength. Specifically, following 50 cycles of sulfate dry-wet exposure, the concrete containing NT exhibited 3.7 % of the mass lost, as opposed to 1.4 % for the unaltered concrete. Meanwhile, the compressive strength of concrete lacking nano-TiO_2_ was reduced by 39.5 %, whereas identical concrete with nano-TiO_2_ showed a lower loss of 35.6 %. By adding 1.0, 3.0, and 5.0 wt percent of titania nanoparticles (NT) to mortar, Daniyal et al. [119] investigated the implications of different concentrations of NT. The resulting mortars were exposed to various atmospheric factors, such as freshwater, salt water, and an alkaline solution with 1 % H_2_SO_4_. After 360 days, the study showed that nano-titanium dioxide greatly increased compressive strength, especially in hard environments like salty and acidic ones. The nano-TiO_2_ composition is closely associated with increases in resisting corrosion and compressive strength with higher quantities producing noticeable advantages. Subsequent analysis was conducted by Shaaban et al. [140] to determine the impact of titania nanomaterials added at 3.0, 6.0, and 9.0 wt percent on mortar resistance to sulfate assault. Their results unveiled that with an increase in the concentration of titania nanoparticles (NT), there was a significant reduction in mass loss. This reduction strongly indicated a notable enhancement in the mortars' ability to withstand sulfate attack. Additionally, the study reported that mortars containing 9.0 wt% NT experienced a shrinkage effect when exposed to a sulfate solution. Martins et al. [114] and Mohseni et al. [115]conducted a study to investigate the influence of nano-titania (NT) on the high-performance concrete resistance to sulfuric acid attack, assessed through tests measuring mass reduction. Their research indicates that the inclusion of 1.0 wt% NT in high-performance concrete can improve its resistance to sulfuric acid attack. However, elevated NT concentrations, such as 3.0 wt%, did not yield the same positive effects and, in some cases, led to increased mass loss compared to the reference concrete. Rahim et al. [113] performed a corrosion test with a 5 wt% NaCl solution and a 5 wt% H_2_SO_4_ solution. It revealed that nano-titania (NT) effectively reduced the corrosion rate induced by H_2_SO_4_ ions among various nanomaterials. Remarkably, the addition of NT resulted in a significant 49.81 % decrease in the corrosion rate. These findings emphasize the superior performance of NT in mitigating H_2_SO_4_-induced corrosion compared to other nanomaterials investigated.

#### 5.3.5 Electrical resistivity

**Fig. 14:**
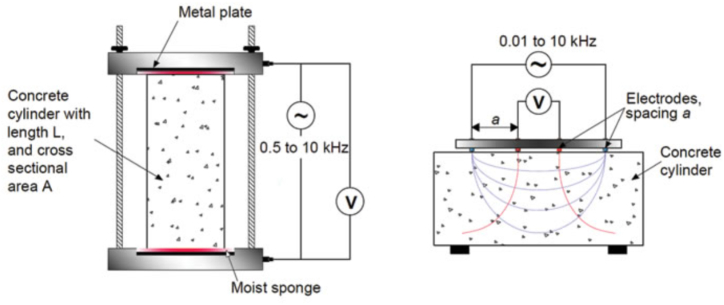
Diagram depicting the procedure for measuring resistivity in concrete after mixing.

The inclusion of nano-TiO_2_ can indeed elevate the electrical resistivity of cement-based materials. Higher electrical resistivity is beneficial for preventing the corrosion of steel reinforcement embedded in concrete structures (Fig. 14). This is because electrical resistivity is closely linked to the porosity and permeability of the material, as permeability diminishes, electrical resistivity rises, thereby enhancing longevity and robustness [110,114,128]. Xiong et al. [145] evaluated the impact of incorporating nano-titania (NT) on the electrical conductivity of cementitious composites. The research illustrated the effective dispersion of Nano-TiO_2_ within the matrix using ultrasonic methods, leading to an augmentation in the electrical conductive properties of cementitious composite materials. Initially, with increasing NT concentration (at levels 3 %, 5 %, and 7 %), there was an enhancement in the electrical conductive characteristics of the cementitious composite materials. However, at higher concentrations of NT, there was a subsequent decrease in electrical conductivity. Notably, the study's highest reported electrical conductivity value was achieved with cement-based materials modified with 5 % Nano-TiO_2_; achieving a value of 17.54 Ω cm. Mohseni et al. [115] observed that specimens of cementitious composite materials with fly ash content displayed an electrical resistivity measuring 7.2 kΩ cm, however, with the addition of 5 wt % of nano-titania (NT), the electrical resistivity increased to 25 kΩ cm. Gopalakrishnan et al. [86] established a direct correlation between the resistivity of mortar and the presence of nano-titanium dioxide (NT). Specifically, it was noted that incorporating 10 wt% NT resulted in a 21 % surge in the electrical resistivity of mortar. Joshaghani et al. [110]investigated the influence of nano-TiO_2_ (NT) on the electrical resistivity of self-compacting concrete (SCC). Their results showed a notable rise in the resistivity of concrete with the incorporation of NT, and these effects were particularly noticeable at 28 and 91 days. Jiang et al. [146]a gradual decrease in electrical resistance that was observed rising with a concentration of nano-titanium dioxide (NT). It was found that resistivity decreased when incorporating 0.1 %, 0.5 %, and 1 % NT into the cement slurry. At 1 % NT content, the resistivity measured only 4.3 × 10^−3^ Ω cm. Zhang et al. [141] have investigated the influence of the crystal structure of nano-titania (NT) on the electrical properties of concrete. His findings demonstrated that adding 5 % anatase nano-TiO_2_ resulted in a 27.36 % decrease in concrete resistivity. This suggests that the reduction in resistivity of cementitious composites due to Nano-TiO_2_ is primarily attributed to the qualities of its semi-conductivity. Additionally, a comprehensive and stable conductive network is established when Nano-TiO_2_ is effectively diffused throughout the cement-based material matrix, refining the pores through a nucleation action that enhances the conductive connection between nano-TiO_2_ particles.

#### 5.3.6 Ultrasonic pulse velocity (UPV)

Ultrasonic Pulse Velocity (UPV) is a valuable metric for assessing the consistency, integrity, and longevity of cement-based materials. A more compact microstructure characterized by reduced pore density leads to a shorter duration of pulse travel time, leading to an increased pulse velocity. Therefore, a higher UPV value signifies superior quality cement-based material with reduced porosity.

**Fig. 15:**
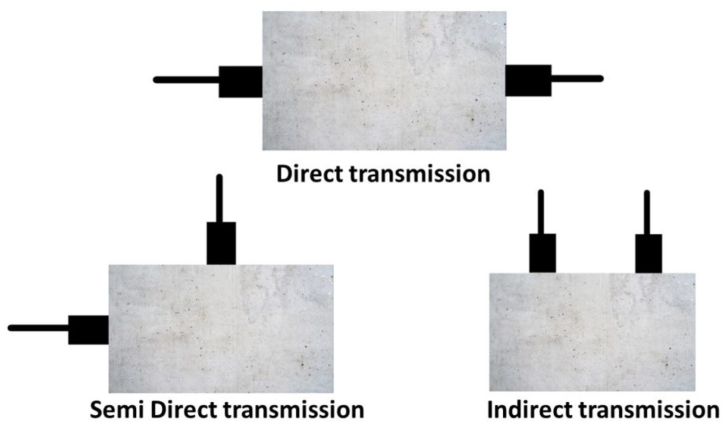
Ultrasonic pulse velocity test.

Multiple reports have shown that introducing nano-titania (NT) enhances the UPV of cement-based materials. This enhancement typically grows as the Nano-TiO_2_ content increases, reaching an optimal level, after which it declines [115,128]. Fig. 15 illustrates the UPV measurement mechanism, conforming to ASTM C597 [147]. Dezhampanah et al. [148] conducted a research investigation to assess the presence of nano-titania (NT), which influences the ultrasonic pulse velocity (UPV) in heavy-weight concrete. The concrete in the study contained 0.6 % polypropylene fibre and varying Nano-TiO_2_ contents of 2.0, 4.0, 6.0, and 8.0 wt%. Their results revealed that incorporating Nano-TiO_2_ until reaching a concentration of 6.0 wt%, led to an increase in UPV. Ultrasonic Pulse Velocity (UPV) decreased when the amount of nano-TiO_2_ was increased above a certain value. Nikbin et al. [149] investigated robust concrete that contained different concentrations of nano-TiO2 (2.0, 4.0, 6.0, and 8.0 wt%) and discovered that UPV elevated with NT content, exceeding 6.0 wt%, where it demonstrated a 15 % improvement over standard concrete. Xu et al. [130] analogously noted that nano-TiO_2_ raised more UPV in concrete exposed to 0–50 sulfate dry-wet cycles. Moreover, Martins et al. [114] showed that 1.0 wt% nano-TiO_2_ was added to outstanding concrete construction performance. Further, with an increase in Nano-TiO_2_ content beyond this threshold, UPV exhibited a decline. The findings also highlighted that incorporating a combination of Nano-TiO_2_ and 30 wt% fly ash yielded notably superior performance compared to NT or fly ash. Garima Rawat et al. [150]the introduction of 1.5 % nano-TiO_2_ improved the uniformity and structural integrity of the resulting concrete, as evidenced by alterations in the Ultrasonic Pulse Velocity (UPV) measurements.

### 5.4 Microstructural properties

Nano-TiO_2_ enhances the microstructure of cementitious composites, leading to denser matrices and impacting the hydration processes. Scanning electron microscope (SEM) images offer insights into nanoparticle distribution, pore enhancement, and forming bonds with the C–S–H gel. Nano-TiO_2_, often referred to as NT, enhances the microstructure of both concrete and mortar by improving pore structure. This reduces specific pore volumes, converting dispersed pores into finer, less detrimental ones. Consequently, these changes lead to increased uniformity, better compaction, decreased porosity, a reduced incidence of micro cracks, and enhanced adhesion facilitating a stronger connection between cement mixture and aggregates, ultimately strengthening the overall performance of the materials [18,105,108,110].

#### 5.4.1 Pore structure

The pore structure is a critical factor for cementitious material strength and durability. Interconnected pores can weaken these materials when filled with air or water, causing fragility, dissolution, or erosion. Conversely, smaller, evenly distributed pores reduce stress concentrations, enhance strength, and hinder substance penetration (Fig. 15). Incorporating zero-dimensional nanoparticles such as nano-TiO_2_ (NT) exploits the "small size effect" and "filling effect," serving as nuclei that impact the pore size of cement-based composites, resulting in denser microstructures and lower permeability. Ma et al. [100], investigated the pore structure of mortar that had undergone a 28-day hydration process was analyzed. The results indicated that the integration of 3 % (NT) significantly improved the pore structure of the mortars. Specifically, there was a 37.7 % increase in moderate pores (those with a size of less than 10 nm), while the number of harmful holes was significantly reduced by 34.6 %. (with a diameter exceeding 50 nm). Moreover, the total specific pore volume in the mortar was decreased by a substantial 48.2 %. These findings suggest that the incorporation of NT positively affects the pore characteristics of the mortar, producing a less porous and refined microstructure. According to Moro et al. [138], cement mixes with water/cement ratios (w/b) of 0.45 and 0.55 had porosity reductions of 5.29 % and 11.66 %, respectively, with the addition of 1 % nano-TiO_2_. At increasing water/cement ratios, the beneficial impact of nano-TiO_2_ on lowering pores was more noticeable, indicating a stronger influence on cement paste properties when there was more water. After examining the nano-TiO_2_ particle size affected the structure of pores, Chen et al. [43]found that bigger NT particles were linked to decreased porosity in cementitious materials. This is probably because smaller fragments tended to aggregate in the slurry. An overabundance of NT can create voids and nanoparticle agglomeration, which raises the total porosity and the number of hazardous pores. Zhang et al. [91], studied by adding nano-TiO_2_ (NT) to cementitious composites, consistently reducing the most probable pore diameter, significantly decreasing from 103 nm to 84 nm at 1 wt % NT, 53 nm at 3 wt % NT, and 47 nm at 5 wt % NT. A reduction in accumulative pore volume accompanied this as NT content increased. Pore size distribution analysis revealed a substantial decrease in critical pore sizes, decreasing by 19.4 %, 48.5 %, and 54.4 % with 1, 3, and 5 wt% Nano-TiO_2_, respectively. These findings affirm the positive impact of Nano-TiO_2_ on improving the quality and performance of cementitious composites. Soleymani et al. [135] documented a decline in the porosity of cement-based composites over time, attributing it to the incorporation of nano-TiO_2_ (NT). Fawzy [151] observed a reduction in capillary porosity, reduced from 5.6 % to 3.8 %, upon introducing 1 wt% nano-TiO_2_ (NT). This signifies an enhancement in the microstructure of cement composites facilitated by the presence of Nano-TiO_2_ particles. Additionally, Li et al. [98] demonstrated that the porosity of reactive powder concrete enhanced with nano-TiO_2_ decreased by 2.08 %. This reduction was determined using a model illustrating how NT enhances compactness, decreasing porosity from 9.04 % to 6.96 %. These findings emphasize NT's role in strengthening cementitious composite microstructure, lowering porosity, and improving material properties.

#### 5.4.2 Impact on hydration products

Nano-TiO_2_ can engage with the hydration of cement, potentially influencing the origins of hydration products and the materials overall microstructure. Throughout the hydration of cement, intricate reactions give rise to diverse products such as C-S-H, CH (calcium hydroxide), and ettringite among others. These resultant compounds, in terms of their type, quantity, and morphology, exhibit variations with the aging of cement. The introduction of nano-TiO_2_ (NT) directly shapes the characteristics of cement-based materials, influencing the generation and attributes of these hydration products [152].

The categories, amounts, and configurations of these products of the hydration process significantly influence the mechanical characteristics of cement-based composites enhanced with NT. In the investigation conducted by Zhang et al. [91]the influence of nano-TiO2 on the structure of the phase and the content of cement products of hydration was investigated. Throughout hydration (3–28 days), a reduction of 8.7 % and 17.7 % was observed in the concentration of tri-calcium silicate (C_3_S) and di-calcium silicate (C_2_S) respectively, in comparison to the standard (cement without Nano-TiO_2_). This decrease in C_3_S and C_2_S content is likely attributed to NT's promotion of C_3_S and C_2_S reaction of hydration. Additionally, the produced calcium hydroxides (CH) diffraction peak strength increased by 50.1 % compared to the standard. It suggested that NT enhances C_3_S and C_2_S of hydration, leading to a notable augmentation in the CH concentration within the cement-based materials. Meng et al. [116]reported a consistent quantity of calcium hydroxide crystal during the initial stages of hydration upon introducing nano-TiO_2_ (NT). However, a discernible shift in the orientation of CH crystal growth was observed. Moreover, the incorporation of 5 % NT resulted in a significant 61 % decrease in the orientation index of initial stage CH crystal growth. This discovery implies that the augmentation of early hydration is not solely attributed to an increase in the quantity of hydration products but is also influenced by alterations within the crystal lattice of the phase in physical terms. In cement mortars containing 3 % nano-TiO_2_ (NT), Ma et al. [100] observed a substantial increase in the amount of ettringite (AFt). The increase was remarkable, with a 50.8 % rise after 3 days and a further increase to 61.6 % after 28 days. Additionally, it was observed that AFt crystals more readily formed upon the exterior of nano-TiO_2_, and it was changed from having a long needle-like structure to having shorter rods or full or partial polyhedral structures. The complex process of increasing AFt development with nano-TiO_2_ involves the interaction of several elements. Nazari et al. [136] investigated the ability of nano-TiO_2_ (NT) to stimulate cement hydration and expedite the process of C-S-H (calcium-silicate-hydrate) gel production. Thus, incorporating Nano-TiO_2_ can improve cementitious composite microstructure, resulting in improved mechanical properties. Notably, in cement paste containing 5 % NT, the intensity of calcium hydroxide (CH) at 28 days raised by 50.1 % as contrasted with the cement paste control. Concurrently, the intensity of C_2_S (dicalcium silicate) and C_3_S (tricalcium silicate) in NT-reinforced cement paste decreased by 17.7 % compared to the control cement paste at 28 days. These observations indicate that NT promotes more efficient hydration, improving mechanical properties and alterations inside the cementitious matrix composition. Additionally, studies by Han et al. [153] demonstrated that nano-TiO_2_ (NT) can control the organization and growth focal point of products formed during cement hydration. Consequently, Nano-TiO_2_ has the potential to promote cement hydration, leading to an increased creation of calcium-silicate-hydrate gel, while simultaneously constraining the expansion of calcium hydroxide. This might lead to a more homogeneous and dense cement binder.

### 5.5 Functional properties

#### 5.5.1 Photocatalytic effect

**Fig. 16:**
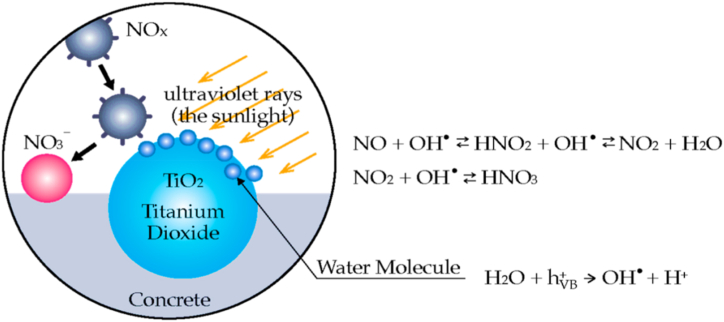
The mechanism of photocatalytic oxidation of NOx.

The photocatalytic effect accelerates chemical reactions through light absorption, typically in the UV or visible spectrum, by a photocatalyst. It enables reactions that wouldn't occur in darkness. Extended exposure to atmospheric contaminants exposes cement-based substances to pollutants that photocatalysts like TiO_2_ may degrade, giving the materials their self-cleaning properties and air-purifying abilities. Photons exciting electrons in the photocatalyst create electron-hole pairs that drive redox reactions, catalyzing chemical processes. Photocatalysis has diverse applications in environmental remediation (air and water purification, self-cleaning surfaces), and energy production (solar cells, hydrogen production). It offers environmental benefits by degrading pollutants in the air and water. TiO_2_, a crucial semiconductor with a 3.2 eV energy bandgap, absorbs light, producing electron-hole pairs that catalyze surface reactions, producing the photocatalytic effect [154].Fig. 16 illustrates the mechanism of photocatalytic oxidation of NOx [155].

Concrete constructions can deteriorate more quickly when substances made from cement are exposed to a wide range of organic and inorganic contaminants over an extended period. As indicated by inquiries [154,[156], [157], [158], [159], [160], [161], [162], [163]], certain investigations have illustrated that some photocatalysts, represented by NT (presuming NT denotes a particular photocatalyst), have notable photocatalytic properties essential for the breakdown of organic substances like dyes and inorganic substances like NOx. This degradation process significantly contributes to the emergence of self-cleansing attributes and air-purifying characteristics within these materials.

#### 5.5.2 Photocatalytic property

Maury-Ramírez et al. [164]suggest that porosity in materials can enhance air purification while hindering self-cleaning performance. This indicates a trade-off in material characteristics when balancing air purification and self-cleaning capabilities. It also demonstrates that roughness adversely affects self-cleaning but does not influence air purification. Victor et al. [165]added different categories of nanomaterials (anatase I, anatase II, and rutile) to mortar and found that these additives could enhance NOx (nitrogen oxide) removal rates under specific conditions, such as UV-A radiation and humidity. Wang et al. [156] studied the effects of different NT concentration levels on the color degradation of solvated NT-treated cementitious materials when exposed to UV irradiation. Higher NT content resulted in more significant color degradation. Saini et al. [158]found that incorporating 3 % NT into cementitious materials led to considerable deterioration of surface MB dyes. Additionally, activated dolomite was essential in improving the capacity of the cement-like substances changed by Nano-TiO_2_ for self-cleaning.

**Fig. 17:**
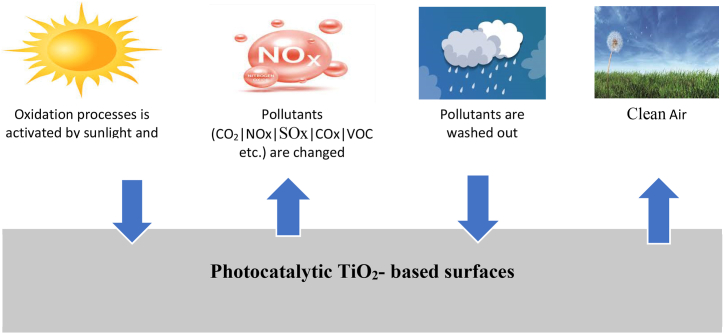
Schematic diagram of Photocatalysis.

Poon and Cheung et al. [166]examined the use of cementitious composites with Nano-TiO_2_ for NOx removal. They found that an optimized blend with 10 % Nano-TiO2 may eliminate NO at 4.01 mg per square meter per hour. Demeestere et al. [167]investigated using Nano-TiO2 as a form of photocatalyst in cementitious composite pavement bricks. Notably, it achieved a high degradation efficiency of organic compounds, and relative humidity and gas flow rates influenced the efficiency. Extending the gas duration of residence in a reaction container was recommended to accelerate deterioration capabilities. M.-Z. Guo et al. [168] manufactured concrete blocks with an exterior layer containing 2 wt% TiO_2_. When exposed to UVA irradiation, these blocks showed a significantly higher photocatalytic removal rate than control samples. The observation of nitrite (NO_2_) and nitrite (NO_3_) generation emerged during the photocatalytic degradation method. Choi et al. [162] showcased that integrating NT into ultra-high-performance concrete significantly reduced the concentration of surface NOx upon exposure to ultraviolet light. A notable 7.7-fold enhancement in the rate of NOx elimination was documented. Utilizing nanomaterial-modified materials holds promise in advancing air purification and self-cleaning attributes, especially in nitrogen oxides and organic compound elimination. Still, the efficacy of these materials is contingent on specific conditions, as well as the category and quantity of nanomaterial incorporated. Beeldens [169] involved subjecting NT-engineered cementitious composite pavement bricks to 53 min of UV irradiation and analyzing the change in NO (nitric oxide) and NOx (nitrogen oxides, including NO and NO_2_) concentrations. The results revealed a minor increase in NO_2_ concentration but a substantial decrease in NOx levels, which includes both NO and NO_2_. This outcome suggests that the photocatalytic process, activated by combining UV irradiation and nano-titanium (NT) in the cementitious composite pavement bricks, facilitated NO transformation into NO_2_ while reducing the overall NOx concentration. When nanomaterials treated with photocatalytic reagents such as TiO_2_ are utilized in air purification procedures, the photocatalytic degradation technique shown in Fig. 17 is extensively employed.

## 6 Conclusion

The major characteristics of nano-titanium dioxide have been the subject of this thorough investigation, with a particular emphasis on how it influences mechanical properties, microstructural changes, and durability of cementitious materials. This research also assessed the possible structural advantages of adding Nano-TiO_2_ to composites that resemble cement. The following is a summary of the primary outcomes.

1. The workability and initial and final setting periods of composites composed of cement are significantly reduced upon the inclusion of nano-titanium dioxide (NT).
2. The mechanical characteristics of cement-based substances can be considerably improved with an ideal concentration of nano-TiO_2_. Mechanical qualities are often improved initially by increasing Nano-TiO_2_ content, but high levels cause a deterioration. The favorable influence of lesser Nano-TiO_2_ nanoparticles on physical properties is more noticeable than that of larger counterparts.
3. Effectively implementing nano-titanium dioxide increases the longevity of cement-like composites by increasing impermeability, carbonation resistance, and corrosion resistance. The optimal quantity of Nano-TiO_2_ is determined by the size of particles and water-to-cement (w/c) proportion.
4. Cementitious composites treated with nano-titanium dioxide exhibit self-cleaning, purifying the atmosphere, and antibacterial characteristics. This study discusses new investigation and implementation concepts for producing useful and economically viable alternatives to typical cementitious substances.

## Prospects for expanded exploration into Nano-TiO₂ engineered cementitious materials

Cementitious composite materials with added nano-TiO_2_ have a lot of promise to improve sustainability and material qualities. Future studies ought to focus on these specific areas of interest.

• Determining the appropriate size of particles, structure, and exterior modifications to enhance efficiency, particularly in conjunction with supplemental cementitious materials (SCMs), as well as the ideal concentrations and combinations of nano-TiO_2_ with other materials.
• Examining the way nano-TiO_2_ modified composite materials function over an extended time in a variety of ambient settings, including variations in humidity, temperature, exposure to chemicals, freezing and thawing cycles, sulfate attacks, chloride penetration, and the carbonation process.
• Long-term performance of nano-titanium dioxide-modified composites is being investigated under various environmental circumstances, including humidity, temperature fluctuations, chemical exposure, freeze-thaw cycles, sulfate attack, chloride penetration, and carbonation.
• Investigating nano-titanium dioxide self-cleaning and photocatalytic characteristics for urban air purification and pollution reduction. Additionally, nano-TiO_2_ can be combined into adaptive construction supplies to respond to exterior triggers like self-treatment and temperature regulation.
• Utilize sophisticated methods like SEM, XRD, and NMR to analyze the microstructural changes caused by TiO2 nanoparticles.
• Performing life cycle assessments (LCA) to study the environmental effect and sustainability of nano-TiO2 in cementitious composites, including energy utilization, ecological footprint, and consumption of resources.
• Contrasting nano-TiO_2_ with other nanomaterials including silica nanoparticles, carbon nanotubes, and graphene oxide to find the best pairings for particular purposes. Field studies should examine the real-world performance of nano-NT modified composites, including instances spanning various domains highlighting tangible advantages and problems.

By addressing these research areas, the construction industry can better leverage nano-TiO_2_ to enhance the performance and sustainability of cementitious composites, leading to more durable, environmentally friendly, and innovative building practices.

## CRediT authorship contribution statement

**J. Jenima:** Writing – review & editing, Writing – original draft, Resources, Methodology, Investigation, Formal analysis, Data curation, Conceptualization. **M. Priya Dharshini:** Writing – review & editing, Writing – original draft, Supervision, Methodology, Investigation, Formal analysis, Data curation, Conceptualization. **M.L. Ajin:** Writing – review & editing, Software, Project administration, Conceptualization. **J. Jebeen Moses:** Writing – review & editing, Resources, Conceptualization. **Krishna Priya Retnam:** Writing – review & editing, Visualization, Validation, Software, Project administration, Methodology, Investigation, Data curation, Conceptualization. **Krishna Prakash Arunachalam:** Writing – review & editing, Writing – original draft, Visualization, Validation, Supervision, Software, Resources, Project administration, Methodology, Investigation, Funding acquisition, Formal analysis, Data curation, Conceptualization. **Siva Avudaiappan:** Writing – review & editing, Writing – original draft, Visualization, Validation, Supervision, Software, Resources, Project administration, Methodology, Investigation, Funding acquisition, Formal analysis, Data curation, Conceptualization. **Ramon Francisco Arrue Munoz:** Writing – review & editing, Validation, Investigation, Formal analysis, Conceptualization.

## Data availability

The data presented in this study are available on request.

## Declaration of competing interest

The authors declare that they have no known competing financial interests or personal relationships that could have appeared to influence the work reported in this paper.

## References

[1] European Union (2011). EU Commission recommendation of 18 October 2011 on the definition of nanomaterial (2011/696/EU). Off. J. Eur. Union.

[2] Fujishima A., Zhang X. (Oct. 2005). Titanium dioxide photocatalysis: present situation and future approaches. Compt. Rendus Chem..

[3] Ajin M.L., Jebeen Moses J., Priya Dharshini M. (2022). Tribological behavior of AA7075 nanohybrid composites at high temperature. Period. Mineral..

[4] Vishaka E.J., Priya Dharshini M., Shally V., Gerardin Jayam Sr. (Dec. 2021). Structural and optical properties of pure NiO nanoparticles and NiO-Mn2O3, NiO-CdO, NiO-Pb2O3, NiO-ZnO nanocomposites. Jordan Journal of Physics.

[5] Ajin M.L., Moses J., Dharshini M.P. (Apr. 2023). Tribological and machining characteristics of AA7075 hybrid composites and optimizing utilizing modified PROMETHEE approach. Mater. Res. Express.

[6] Vishaka E.J., Priya Dharshini M., Shally V., Gerardin Jayam Sr. (2022). NiO-CdO nanocomposite for photocatalytic applications. Mater Today Proc.

[7] Carp O., Huisman C.L., Reller A. (2004). Photoinduced reactivity of titanium dioxide. Prog. Solid State Chem..

[8] Cassar L. (May 2004). Photocatalysis of cementitious materials: clean buildings and clean air. MRS Bull..

[9] Chen J., Poon C. (Sep. 2009). Photocatalytic construction and building materials: from fundamentals to applications. Build. Environ..

[10] Guo S., Wu Z., Zhao W. (Apr. 2009). TiO2-based building materials: above and beyond traditional applications. Sci. Bull..

[11] Chen F., Yang X., Mak H.K.C., Chan D.W.T. (Aug. 2010). Photocatalytic oxidation for antimicrobial control in built environment: a brief literature overview. Build. Environ..

[12] Maury A., De Belie N. (Jun. 2010). State of the art of TiO2 containing cementitious materials: self-cleaning properties. Mater. de Construction.

[13] Xu Lei (Jan. 2023). Investigations on the rehydration of recycled blended SCMs cement. Cem Concr Res.

[14] Han B., Sun S., Ding S., Zhang L., Yu X., Ou J. (Mar. 2015). Review of nanocarbon-engineered multifunctional cementitious composites. Compos Part A Appl Sci Manuf.

[15] Konsta-Gdoutos M.S., Metaxa Z.S., Shah S.P. (Jul. 2010). Highly dispersed carbon nanotube reinforced cement based materials. Cem Concr Res.

[16] Sikora P., Łukowski P., Cendrowski K., Horszczaruk E., Mijowska E. (2015). The effect of nanosilica on the mechanical properties of polymer-cement composites (PCC). Procedia Eng..

[17] Mulenga D.M., Robery P.C. (May 2010). Structures Congress.

[18] Noorvand H., Abang Ali A.A., Demirboga R., Farzadnia N., Noorvand H. (Oct. 2013). Incorporation of nano TiO2 in black rice husk ash mortars. Constr Build Mater.

[19] Biricik H., Sarier N. (May 2014). Comparative study of the characteristics of nano silica - , silica fume - and fly ash - incorporated cement mortars. Mater. Res..

[20] Lazaro A., Yu Q.L., Brouwers H.J.H. (2016). Sustainability of Construction Materials.

[21] Zhang C.-Y., Han R., Yu B., Wei Y.-M. (May 2018). Accounting process-related CO2 emissions from global cement production under Shared Socioeconomic Pathways. J. Clean. Prod..

[22] Yu R., Tang P., Spiesz P., Brouwers H.J.H. (Jun. 2014). A study of multiple effects of nano-silica and hybrid fibres on the properties of Ultra-High Performance Fibre Reinforced Concrete (UHPFRC) incorporating waste bottom ash (WBA). Constr Build Mater.

[23] Nima F., Ali A.A.A., Demirboga R. (Oct. 2011). Development of nanotechnology in high performance concrete. Adv Mat Res.

[24] Kewalramani M.A., Syed Z.I. (May 2018). Application of nanomaterials to enhance microstructure and mechanical properties of concrete. International Journal of Integrated Engineering.

[25] Siler P. (Jul. 2019). The effect of zinc, water to binder ratio and silica fume on the hydration and mechanical properties of Portland cement mixtures. IOP Conf. Ser. Mater. Sci. Eng..

[26] Patil A.V. (2024). Performance analysis of self-compacting concrete with use of artificial aggregate and partial replacement of cement by fly ash. Buildings.

[27] Seddik Meddah M. (Mar. 2015). Durability performance and engineering properties of shale and volcanic ashes concretes. Constr Build Mater.

[28] Reches Y. (Jun. 2018). Nanoparticles as concrete additives: review and perspectives. Constr Build Mater.

[29] Vishwakarma V., Ramachandran D. (Feb. 2018). Green Concrete mix using solid waste and nanoparticles as alternatives – a review. Constr Build Mater.

[30] Kong D., Huang S., Corr D., Yang Y., Shah S.P. (Mar. 2018). Whether do nano-particles act as nucleation sites for C-S-H gel growth during cement hydration. Cem. Concr. Compos..

[31] Mohan A., Priya R.K., Arunachalam K.P., Avudaiappan S., Maureira-Carsalade N., Roco-Videla A.G. (2023). Investigating the mechanical, thermal, and crystalline properties of raw and potassium hydroxide treated butea parviflora fibers for green polymer composites. Polymers.

[32] Sheeba K.R.J., Priya R.K., Arunachalam K.P., Avudaiappan S., Saavedra Flores E.I., Kozlov P.A. (2023). Enhancing structural, thermal, and mechanical properties of Acacia pennata natural fibers through benzoyl chloride treatment for construction applications. Case Stud. Constr. Mater..

[33] Arunachalam K.P., Henderson J.H. (2023). Experimental study on mechanical strength of vibro-compacted interlocking concrete blocks using image processing and microstructural analysis. Iranian Journal of Science and Technology - Transactions of Civil Engineering.

[34] Han B., Ding S., Wang J., Ou J. (2019).

[35] Raki L., Beaudoin J., Alizadeh R., Makar J., Sato T. (Feb. 2010). Cement and concrete nanoscience and nanotechnology. Materials.

[36] P. Kumar, F. Azarmi, and M. Mulheron, “Enlightening and noxious shades of nanotechnology application in concrete,” Nanotechnology: Volume 7 Civil/Construction Engineering. (Studium Press LLC, USA; Govil, J.N. Eds.). ISBN: 1- 62699-009-3. pp. 255-287.

[37] Sun Y., Zhang P., Guo J., Wang L., Wu J. (Apr. 2024). Rheological properties and workability of PVA fiber and nano-SiO2 modified cement-based materials. Developments in the Built Environment.

[38] Zhang P., Li X., Guo J., Gao Z. (Oct. 2024). Fracture properties of cementitious composites containing nano-materials: a comprehensive review. Theor. Appl. Fract. Mech..

[39] Zhang X., Zhang P., Yuan W., Hu S. (Mar. 2024). Durability prediction of geopolymer mortar reinforced with nanoparticles and PVA fiber using particle swarm optimized BP neural network. Nanotechnol. Rev..

[40] Zhang P., Sun Y., Wu J., Hong J., Gao Z. (Dec. 2023). Mechanical properties and microstructure of nano-modified geopolymer concrete containing hybrid fibers after exposure to elevated temperature. Constr Build Mater.

[41] Nazari A., Riahi S., Fatemeh Shamekhi S., Khademno A. (2010). Assessment of the effects of the cement paste composite in presence TiO2 nanoparticles. J. Am. Sci..

[42] Pacheco-Torgal F., Jalali S. (Feb. 2011). Nanotechnology: advantages and drawbacks in the field of construction and building materials. Constr Build Mater.

[43] Chen J., Kou S., Poon C. (May 2012). Hydration and properties of nano-TiO2 blended cement composites. Cem. Concr. Compos..

[44] Janczarek M., Klapiszewski Ł., Jędrzejczak P., Klapiszewska I., Ślosarczyk A., Jesionowski T. (Feb. 2022). Progress of functionalized TiO2-based nanomaterials in the construction industry: a comprehensive review. Chem. Eng. J..

[45] Fujishima A., Honda K. (Jul. 1972). Electrochemical photolysis of water at a semiconductor electrode. Nature.

[46] Kavitha S.A., Priya R.K., Arunachalam K.P., Avudaiappan S., Saavedra Flores E.I., Blanco D. (2024). Experimental investigation on strengthening of Zea mays root fibres for biodegradable composite materials using potassium permanganate treatment. Sci. Rep..

[47] Rekha M.S., Raju S., Arunachalam K.P., Avudaiappan S., Abbas M.A.E., Fernande D.B. (2024). Effects of alkaline concentration on workability and strength properties of ambient cured green geopolymer concrete. Asian Journal of Civil Engineering.

[48] Sheeba K.R.J., Priya R.K., Arunachalam K.P., Shobana S., Avudaiappan S., Saavedra Flores E.I. (2023). Examining the physico-chemical, structural and thermo-mechanical properties of naturally occurring Acacia pennata fibres treated with KMnO_4_. Sci. Rep..

[49] Avudaiappan S. (2023). Experimental investigation on the physical, microstructural, and mechanical properties of hemp limecrete. Sci. Rep..

[50] Bunea G., Alexa-Stratulat S.-M., Mihai P., Toma I.-O. (Feb. 2023). Use of clay and titanium dioxide nanoparticles in mortar and concrete—a state-of-the-art analysis. Coatings.

[51] Siang Ng D. (Oct. 2020). Influence of SiO2, TiO2 and Fe2O3 nanoparticles on the properties of fly ash blended cement mortars. Constr Build Mater.

[52] W. Tang, L. Jiang, and Z. Chena, “Review on photocatalytic activity of TiO2 nanoparticles and their synthesized methods,” Mater. Today Chem., 18, 100361.

[53] Huang S., Chueh P.J., Lin Y.-W., Shih T.-S., Chuang S.-M. (Dec. 2009). Disturbed mitotic progression and genome segregation are involved in cell transformation mediated by nano-TiO2 long-term exposure. Toxicol. Appl. Pharmacol..

[54] Song W. (2013). Titanium dioxide nanoparticles induced proinflammation of primary cultured cardiac myocytes of rat. J. Nanomater..

[55] Macwan D.P., Dave P.N., Chaturvedi S. (Jun. 2011). A review on nano-TiO2 sol–gel type syntheses and its applications. J. Mater. Sci..

[56] Devasena M., Sangeetha V. (Dec. 2021). Implications of nano-titanium dioxide incorporation in cement matrix: a review. J. Inst. Eng.: Series D.

[57] Robichaud C.O., Uyar A.E., Darby M.R., Zucker L.G., Wiesner M.R. (Jun. 2009). Estimates of upper bounds and trends in nano-TiO _2_ production as a basis for exposure assessment. Environ. Sci. Technol..

[58] Verma R., Mantri B., Kumar Srivastava A. (Apr. 2015). “ shape control synthesis, characterizations, mechanisms and optical properties of larg scaled metal oxide nanostructures of ZnO and TiO2,”. Adv. Mater. Lett..

[59] Meacock G., Taylor K.D.A., Knowles M.J., Himonides A. (Feb. 1997). The improved whitening of minced cod flesh using dispersed titanium dioxide. J. Sci. Food Agric..

[60] Patel N., Mishra C.B. (May 2018). Laboratory Investigation of nano titanium dioxide (TiO2) in concrete for pavement. International Research Journal of Engineering and Technology (IRJET).

[61] Luo G., Liu H., Li W., Lyu X. (Oct. 2020). Automobile exhaust removal performance of pervious concrete with nano TiO2 under photocatalysis. Nanomaterials.

[62] Reyes-Coronado D., Rodríguez-Gattorno G., Espinosa-Pesqueira M.E., Cab C., de Coss R., Oskam G. (Apr. 2008). Phase-pure TiO _2_ nanoparticles: anatase, brookite and rutile. Nanotechnology.

[63] Fujishima A., Zhang X., Tryk D. (Sep. 2007). Heterogeneous photocatalysis: from water photolysis to applications in environmental cleanup. Int. J. Hydrogen Energy.

[64] Addamo M., Bellardita M., Di Paola A., Palmisano L. (2006). Preparation and photoactivity of nanostructured anatase, rutile and brookite TiO2 thin films. Chem. Commun..

[65] Bakardjieva S. (2006). Transformation of brookite-type TiO2 nanocrystals to rutile: correlation between microstructure and photoactivity. J. Mater. Chem..

[66] Hamidi F., Aslani F. (Oct. 2019). TiO2-based photocatalytic cementitious composites: materials, properties, influential parameters, and assessment techniques. Nanomaterials.

[67] Mohamed H. (Sep. 2020). Mechanical and microstructural properties of geopolymer mortars from meta-halloysite: effect of titanium dioxide TiO2 (anatase and rutile) content. SN Appl. Sci..

[68] Park J.H., Kim S., Bard A.J. (Jan. 2006). Novel carbon-doped TiO _2_ nanotube arrays with high aspect ratios for efficient solar water splitting. Nano Lett..

[69] Nazeeruddin M.K. (Jul. 1993). Conversion of light to electricity by cis-X2bis(2,2’-bipyridyl-4,4’-dicarboxylate) ruthenium(II) charge-transfer sensitizers (X = Cl-, Br-, I-, CN-, and SCN-) on nanocrystalline titanium dioxide electrodes. J. Am. Chem. Soc..

[70] Parkin I.P., Palgrave R.G. (2005). Self-cleaning coatings. J. Mater. Chem..

[71] Lackhoff M., Prieto X., Nestle N., Dehn F., Niessner R. (Jul. 2003). Photocatalytic activity of semiconductor-modified cement—influence of semiconductor type and cement ageing. Appl. Catal., B.

[72] Hüsken G., Hunger M., Ballari M.M., Brouwers H.J.H. (2009). Nanotechnology in Construction 3.

[73] Ballari M.M., Hunger M., Hüsken G., Brouwers H.J.H. (2009). Nanotechnology in Construction 3.

[74] Fujishima A., Hashimoto K., Watanabe (1999).

[75] Sanchez F., Sobolev K. (Nov. 2010). Nanotechnology in concrete – a review. Constr Build Mater.

[76] Linsebigler A.L., Lu G., Yates J.T. (May 1995). Photocatalysis on TiO2 surfaces: principles, mechanisms, and selected results. Chem Rev.

[77] Fujishima A., Rao T.N., Tryk D.A. (Jun. 2000). Titanium dioxide photocatalysis. J. Photochem. Photobiol. C Photochem. Rev..

[78] Martha S., Chandra Sahoo P., Parida K.M. (2015). An overview on visible light responsive metal oxide based photocatalysts for hydrogen energy production. RSC Adv..

[79] Grande F., Tucci P. (Apr. 2016). Titanium dioxide nanoparticles: a risk for human health?. Mini-Rev. Med. Chem..

[80] Poulopoulos S.G., Yerkinova A., Ulykbanova G., Inglezakis V.J. (May 2019). Photocatalytic treatment of organic pollutants in a synthetic wastewater using UV light and combinations of TiO2, H2O2 and Fe(III). PLoS One.

[81] Khdary N.H. (Aug. 2020). Synthesis of superior visible-light-driven nanophotocatalyst using high surface area TiO2 nanoparticles decorated with CuxO particles. Catalysts.

[82] Bianchi C.L., Pirola C., Stucchi M., Sacchi B., Cerrato G., Morandi S., Di Michele A., Carletti A., Capucci V., Cao Wenbin (2016). Semiconductor Photocatalysis-Materials, Mechanisms and Applications.

[83] Atzl B.A., Pupp M., Rupprich M. (Nov. 2018). The use of photocatalysis and titanium dioxide on diesel exhaust fumes for NOx reduction. Sustainability.

[84] Kang X., Liu S., Dai Z., He Y., Song X., Tan Z. (Feb. 2019). Titanium dioxide: from engineering to applications. Catalysts.

[85] Lucas S.S., Ferreira V.M., de Aguiar J.L.B. (Jan. 2013). Incorporation of titanium dioxide nanoparticles in mortars — influence of microstructure in the hardened state properties and photocatalytic activity. Cem Concr Res.

[86] Gopalakrishnan R., Vignesh B., Jeyalakshmi R. (Apr. 2020). Mechanical, electrical and microstructural studies on nano-TiO _2_ admixtured cement mortar cured with industrial wastewater. Engineering Research Express.

[87] Wang L., Zhang H., Gao Y. (Sep. 2018). Effect of TiO _2_ nanoparticles on physical and mechanical properties of cement at low temperatures. Adv. Mater. Sci. Eng..

[88] Ma B., Li H., Li X., Mei J., Lv Y. (Sep. 2016). Influence of nano-TiO2 on physical and hydration characteristics of fly ash–cement systems. Constr Build Mater.

[89] Maury-Ramirez A., Demeestere K., De Belie N. (Apr. 2012). Photocatalytic activity of titanium dioxide nanoparticle coatings applied on autoclaved aerated concrete: effect of weathering on coating physical characteristics and gaseous toluene removal. J. Hazard Mater..

[90] Lee B.Y., Jayapalan A.R., Kurtis K.E. (Nov. 2013). Effects of nano-TiO _2_ on properties of cement-based materials. Mag. Concr. Res..

[91] Zhang R., Cheng X., Hou P., Ye Z. (Apr. 2015). Influences of nano-TiO2 on the properties of cement-based materials: hydration and drying shrinkage. Constr Build Mater.

[92] Liang X., Cui S., Li H., Abdelhady A., Wang H., Zhou H. (Aug. 2019). Removal effect on stormwater runoff pollution of porous concrete treated with nanometer titanium dioxide. Transp Res D Transp Environ.

[93] Yuenyongsuwan J., Sinthupinyo S., O'Rear E.A., Pongprayoon T. (Feb. 2019). Hydration accelerator and photocatalyst of nanotitanium dioxide synthesized via surfactant-assisted method in cement mortar. Cem. Concr. Compos..

[94] Staub de Melo J.V., Trichês G. (Jul. 2018). Study of the influence of nano-TiO _2_ on the properties of Portland cement concrete for application on road surfaces. Road Mater. Pavement Des..

[95] Reches Y., Thomson K., Helbing M., Kosson D.S., Sanchez F. (Apr. 2018). Agglomeration and reactivity of nanoparticles of SiO2, TiO2, Al2O3, Fe2O3, and clays in cement pastes and effects on compressive strength at ambient and elevated temperatures. Constr Build Mater.

[96] Paul S.C., van Rooyen A.S., van Zijl G.P.A.G., Petrik L.F. (Nov. 2018). Properties of cement-based composites using nanoparticles: a comprehensive review. Constr Build Mater.

[97] Silvestro L., Jean Paul Gleize P. (Dec. 2020). Effect of carbon nanotubes on compressive, flexural and tensile strengths of Portland cement-based materials: a systematic literature review. Constr Build Mater.

[98] Li Z., Ding S., Yu X., Han B., Ou J. (Aug. 2018). Multifunctional cementitious composites modified with nano titanium dioxide: a review. Compos Part A Appl Sci Manuf.

[99] Jayapalan A.R., Lee B.Y., Kurtis K.E. (Feb. 2013). Can nanotechnology be ‘green’? Comparing efficacy of nano and microparticles in cementitious materials. Cem. Concr. Compos..

[100] Ma B., Li H., Mei J., Li X., Chen F. (2015). Effects of nano-TiO _2_ on the toughness and durability of cement-based material. Adv. Mater. Sci. Eng..

[101] Salemi N., Behfarn K., Zaree S.A. (2014). Effect of nanoparticles on frost durability of concrete. Asian Journal of Civil Engineering.

[102] Zhang M., Li H. (Feb. 2011). Pore structure and chloride permeability of concrete containing nano-particles for pavement. Constr Build Mater.

[103] Behfarnia K., Azarkeivan A., Keivan A. (2013). The effects of TiO2 and ZnO nanoparticles on physical and mechanical properties of normal concrete. Asian J. of Civil Engineering.

[104] Ren J., Lai Y., Gao J. (Jun. 2018). Exploring the influence of SiO2 and TiO2 nanoparticles on the mechanical properties of concrete. Constr Build Mater.

[105] Nazari A., Riahi S. (Apr. 2011). TiO2 nanoparticles effects on physical, thermal and mechanical properties of self compacting concrete with ground granulated blast furnace slag as binder. Energy Build..

[106] Rawat G., Gandhi S., Murthy Y.I. (Dec. 2022). Strength and rheological aspects of concrete containing nano-titanium dioxide. Asian Journal of Civil Engineering.

[107] Qian G., Yu H., Gong X., Zhao L. (Sep. 2019). Impact of Nano-TiO2 on the NO2 degradation and rheological performance of asphalt pavement. Constr Build Mater.

[108] Jalal M., Fathi M., Farzad M. (Jul. 2013). Effects of fly ash and TiO2 nanoparticles on rheological, mechanical, microstructural and thermal properties of high strength self compacting concrete. Mech. Mater..

[109] Jayapalan A.R., Lee B.Y., Kurti K.E. (2009). Effect of nano-sized titanium dioxide on early age hydration of Portland cement. Nanotechnology in Construction.

[110] Joshaghani A., Balapour M., Mashhadian M., Ozbakkaloglu T. (Jun. 2020). Effects of nano-TiO2, nano-Al2O3, and nano-Fe2O3 on rheology, mechanical and durability properties of self-consolidating concrete (SCC): an experimental study. Constr Build Mater.

[111] Li Z. (Sep. 2017). Effect of nano-titanium dioxide on mechanical and electrical properties and microstructure of reactive powder concrete. Mater. Res. Express.

[112] Han B. (Sep. 2017). Reactive powder concrete reinforced with nano SiO2-coated TiO2. Constr Build Mater.

[113] Nair S.R., Rahim A., Rahim A., Nair S.R. (2016). Influence of nano-materials in high strength concrete. J. Chem. Pharmaceut. Sci..

[114] Martins TM. da R., Pacheco-Torgal F., Miraldo S., Aguiar J.L., Jesus C.M.G. (2016). An experimental investigation on nano-TiO2 and fly ash based high performance concrete. Indian Concr. J..

[115] Mohseni E., Miyandehi B.M., Yang J., Yazdi M.A. (Jun. 2015). Single and combined effects of nano-SiO2, nano-Al2O3 and nano-TiO2 on the mechanical, rheological and durability properties of self-compacting mortar containing fly ash. Constr Build Mater.

[116] Meng T., Yu Y., Qian X., Zhan S., Qian K. (Apr. 2012). Effect of nano-TiO2 on the mechanical properties of cement mortar. Constr Build Mater.

[117] Ma B., Li H., Li X., Mei J., Lv Y. (2016). Influence of nano-TiO2 on physical and hydration characteristics of fly ash–cement systems. Constr. Build. Mater..

[118] Janus M., Mądraszewski S., Zając K., Kusiak-Nejman E., Morawski A.W., Stephan D. (Nov. 2019). Photocatalytic activity and mechanical properties of cements modified with TiO2/N. Materials.

[119] Daniyal M., Akhtar S., Azam A. (Nov. 2019). Effect of nano-TiO2 on the properties of cementitious composites under different exposure environments. J. Mater. Res. Technol..

[120] Arunachalam K.P. (2024).

[121] Nagarajan D. (2023). Experimental and numerical investigations of laced built-up lightweight concrete encased columns subjected to cyclic axial load. Buildings.

[122] Kadhim M.J., Al-Jadiri R.S., AL Wahab Ali M.A. (May 2019). Study the effect of addition nano-TiO _2_ by dispersion method on the some mechanical properties and durability of cement mortar. IOP Conf. Ser. Mater. Sci. Eng..

[123] Farzadnia N., Abang Ali A.A., Demirboga R., Anwar M.P. (Jun. 2013). Characterization of high strength mortars with nano Titania at elevated temperatures. Constr Build Mater.

[124] J. Sorathiya, S. Shah, and S. Kacha, “Effect on Addition of Nano "Titanium Dioxide” (TiO2) on Compressive Strength of Cementitious Concrete,” pp. 219–211. doi: 10.29007/sq9d.

[125] Ying J., Zhou B., Xiao J. (Sep. 2017). Pore structure and chloride diffusivity of recycled aggregate concrete with nano-SiO2 and nano-TiO2. Constr Build Mater.

[126] Nazari A., Riahi S. (2010). The effect of TiO2 nanoparticles on water permeability and thermal and mechanical properties of high strength self compacting concrete. Mater. Sci. Eng., A.

[127] Salman M.M., Eweed K.M., Hameed A.M., Salman M.M., Eweed K.M., Hameed A.M. (2017). Influence of partial replacement TiO2 nanoparticles on the compressive and flexural strength of ordinary cement mortar,”. Al-Nahrain Journal for Engineering Sciences.

[128] Mohseni E., Naseri F., Amjadi R., Khotbehsara M.M., Ranjbar M.M. (Jul. 2016). Microstructure and durability properties of cement mortars containing nano-TiO2 and rice husk ash. Constr Build Mater.

[129] Chunping G., Qiannan W., Jintao L., Wei S. (2018). Effect of nano-TiO₂ on the durability of ultra-high performance concrete with and without a flexural load. Ceramics.

[130] Xu C., Liao H.-H., Chen Y.-L., Du X., Peng B., Fernandez-Steeger T.M. (Oct. 2021). Corrosion performance of nano-TiO2-modified concrete under a dry–wet sulfate environment. Materials.

[131] Tang S.W., Yao Y., Andrade C., Li Z.J. (Dec. 2015). Recent durability studies on concrete structure. Cem Concr Res.

[132] Givi A.N., Rashid S.A., Aziz F.N.A., Salleh M.A.M. (2011). The effects of lime solution on the properties of SiO2 nanoparticles binary blended concrete. Compos. B Eng..

[133] He X., Shi X. (Jan. 2008). Chloride permeability and microstructure of Portland cement mortars incorporating nanomaterials. Transport. Res. Rec.: J. Transport. Res. Board.

[134] Arif M., Al-Hagri M.G., Shariq M., Rahman I., Hassan A., Baqi A. (Mar. 2020). Mechanical properties and microstructure of micro- and nano-additives-based modified concrete composites: a sustainable solution. J. Inst. Eng.: Series A.

[135] Soleymani F. (2012). Assessments of the effects of limewater on water permeability of TiO2 nanoparticles binary blended palm oil clinker aggregate-based concrete. Journal of American Science.

[136] Nazari A., Riahi S. (Nov. 2011). TiO2 nanoparticles effects on properties of concrete using ground granulated blast furnace slag as binder. Sci. China Technol. Sci..

[137] Ramachandran D., Uthaman S., Vishwakarma V. (Aug. 2020). Studies of carbonation process in nanoparticles modified fly ash concrete. Constr Build Mater.

[138] Moro C., El Fil H., Francioso V., Velay-Lizancos M. (Jan. 2021). Influence of water-to-binder ratio on the optimum percentage of nano-TiO2 addition in terms of compressive strength of mortars: a laboratory and virtual experimental study based on ANN model. Constr Build Mater.

[139] Rao S., Silva P., de Brito J. (Oct. 2015). Experimental study of the mechanical properties and durability of self-compacting mortars with nano materials (SiO2 and TiO2). Constr Build Mater.

[140] Shaaban I., Ei-Sayad H., El-Ghaly A., Moussa S. (Jun. 2020). Effect of micro TiO2 on cement mortar. European Journal of Materials Science and Engineering.

[141] Zhang Xiaoyi (2021). Preparation of nano-TiO2 modified cement concrete and its mechanical and durability performance. Met. Funct. Mater..

[142] Duan P., Yan C., Luo W., Zhou W. (Mar. 2016). Effects of adding nano-TiO2 on compressive strength, drying shrinkage, carbonation and microstructure of fluidized bed fly ash based geopolymer paste. Constr Build Mater.

[143] Yu C., Sun W., Scrivener K. (Jan. 2013). Mechanism of expansion of mortars immersed in sodium sulfate solutions. Cem Concr Res.

[144] Müllauer W., Beddoe R.E., Heinz D. (Oct. 2013). Sulfate attack expansion mechanisms. Cem Concr Res.

[145] Xiong G.X., Deng M., Xu L.L., Tang M.S. (2006). Properties of cement-based composites by doping nano-TiO2. J. Chin. Ceram. Soc..

[146] Jiang S. (Jan. 2018). Comparison of compressive strength and electrical resistivity of cementitious composites with different nano- and micro-fillers. Arch. Civ. Mech. Eng..

[147] ASTM C597-16 (2016).

[148] Dezhampanah S., Nikbin I.M., Mehdipour S., Mohebbi R., Moghadam H. (Dec. 2021). Fiber- reinforced concrete containing nano - TiO2 as a new gamma-ray radiation shielding materials. J. Build. Eng..

[149] Nikbin I.M., Mohebbi R., Dezhampanah S., Mehdipour S., Mohammadi R., Nejat T. (Sep. 2019). Gamma ray shielding properties of heavy-weight concrete containing Nano-TiO2. Radiat. Phys. Chem..

[150] Rawat Garima, Gandhi Sumit, Murthy Yogesh Iyer (2023). “ Durability aspects of concrete containing nano titanium dioxide,”. ACI Mater. J..

[151] Fawzy Y.A. (2016). Effect of nano-titanium on properties of concrete made with various cement types. Journal of American Science.

[152] Thomas J.J., Jennings H.M., Chen J.J. (Mar. 2009). Influence of nucleation seeding on the hydration mechanisms of tricalcium silicate and cement. J. Phys. Chem. C.

[153] Han B. (Apr. 2017). Nano-core effect in nano-engineered cementitious composites. Compos Part A Appl Sci Manuf.

[154] Kumar S., Jain S., Yadav Lamba B., Kumar P. (Jan. 2018). Epigrammatic status and perspective of sequestration of carbon dioxide: role of TiO2 as photocatalyst. Sol. Energy.

[155] Devahasdin S., Fan C., Li K., Chen D.H. (Mar. 2003). TiO2 photocatalytic oxidation of nitric oxide: transient behavior and reaction kinetics. J. Photochem. Photobiol. Chem..

[156] Wang Z., Yu Q., Gauvin F., Feng P., Qianping R., Brouwers H.J.H. (Oct. 2020). Nanodispersed TiO2 hydrosol modified Portland cement paste: the underlying role of hydration on self-cleaning mechanisms. Cem Concr Res.

[157] Wang Z., Yu Q., Feng P., Brouwers H.J.H. (Aug. 2022). Variation of self-cleaning performance of nano-TiO2 modified mortar caused by carbonation: from hydrates to carbonates. Cem Concr Res.

[158] Saini A., Ratan J.K. (May 2022). Formulation and evaluation of surface-fluorinated microsized-TiO2 based self-cleaning cement: characterization, self-cleaning, depollution and antimicrobial study. Chem. Pap..

[159] Ratan J.K., Saini A., Verma P. (Sep. 2018). Microsized-titanium dioxide based self-cleaning cement: incorporation of calcined dolomite for enhancement of photocatalytic activity. Mater. Res. Express.

[160] Tung W.S., Daoud W.A. (2011). Self-cleaning fibers via nanotechnology: a virtual reality. J. Mater. Chem..

[161] Shi C., Liu M., He P., Ou Z. (Jun. 2012). Factors affecting kinetics of CO _2_ curing of concrete. J Sustain Cem Based Mater.

[162] Choi H.-J., Park J.-J., Yoo D.-Y. (May 2021). Benefits of TiO2 photocatalyst on mechanical properties and nitrogen oxide removal of ultra-high-performance concrete. Constr Build Mater.

[163] Folli A., Pade C., Hansen T.B., De Marco T., Macphee D.E. (Mar. 2012). TiO2 photocatalysis in cementitious systems: insights into self-cleaning and depollution chemistry. Cem Concr Res.

[164] Maury-Ramírez A. (2011).

[165] Staub de Melo J.V., Trichês G. (Jul. 2018). Study of the influence of nano-TiO _2_ on the properties of Portland cement concrete for application on road surfaces. Road Mater. Pavement Des..

[166] Poon C.S., Cheung E. (Aug. 2007). NO removal efficiency of photocatalytic paving blocks prepared with recycled materials. Constr Build Mater.

[167] Demeestere K., Dewulf J., De Witte B., Beeldens A., Van Langenhove H. (Apr. 2008). Heterogeneous photocatalytic removal of toluene from air on building materials enriched with TiO2. Build. Environ..

[168] Guo M.-Z., Ling T.-C., Poon C.-S. (Feb. 2013). Nano-TiO2-based architectural mortar for NO removal and bacteria inactivation: influence of coating and weathering conditions. Cem. Concr. Compos..

[169] Boonen E., Beeldens A. (Jul. 2014). Recent photocatalytic applications for air purification in Belgium. Coatings.

